# Lipid-based nanoformulations in onychomycosis therapy: addressing challenges of current therapies and advancing treatment

**DOI:** 10.1039/d5ra00387c

**Published:** 2025-03-11

**Authors:** Shiv Kumar Prajapati, Ankit Jain, Meenakshi Bajpai

**Affiliations:** a Institute of Pharmaceutical Research, GLA University Mathura India; b Department of Pharmacy, Birla Institute of Technology and Science-Pilani Pilani Campus Pilani India ankit.j@pilani.bits-pilani.ac.in

## Abstract

Onychomycosis significantly impacts approximately 20% of the global population. The physical barriers of the nail structure make fungal infections a persistent therapeutic challenge. Traditional approaches, including topical and oral antifungal agents, have limitations such as toxicities, low nail permeability, adverse effects, and high recurrence rates. Consequently, emerging lipid-based delivery systems have gained interest because of their potential to address these drawbacks. Nanostructured lipid carriers (NLCs), solid lipid nanoparticles (SLNs), liposomes, and transferosomes are innovative formulations that offer enhanced drug solubility, sustained release, and targeted delivery to the nail matrix. These lipid-mediated approaches have shown promise in overcoming the hurdles associated with conventional therapies, thereby improving treatment outcomes, patient compliance, and the overall quality of life. A comprehensive review focusing on the potential of lipid-based drug delivery systems in treating onychomycosis is lacking in the existing literature. This review explores various aspects of the clinical presentation of onychomycosis, available treatments, challenges associated with treatment, formulation science related to lipid-based vehicles and their applications, highlighted by the promising aspects of these novel formulations, and provides insights into clinical developments. In addition, the regulatory perspective is critical to such development, and approval is discussed, particularly in managing regulatory compliance complexities to ensure successful implementation. The holistic approach provides a comprehensive basis for determining lipid-based drug delivery systems' state-of-the-art and future scope.

## Abstract 2

Onychomycosis significantly impacts approximately 20% of the global population.

## Graphical abstract

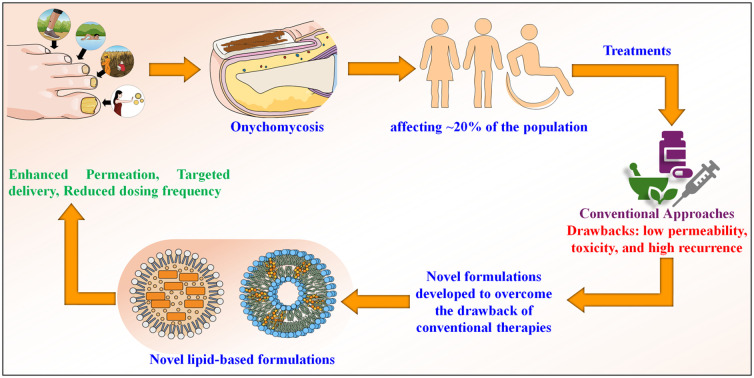

## 1. Introduction

**Fig. 1:**
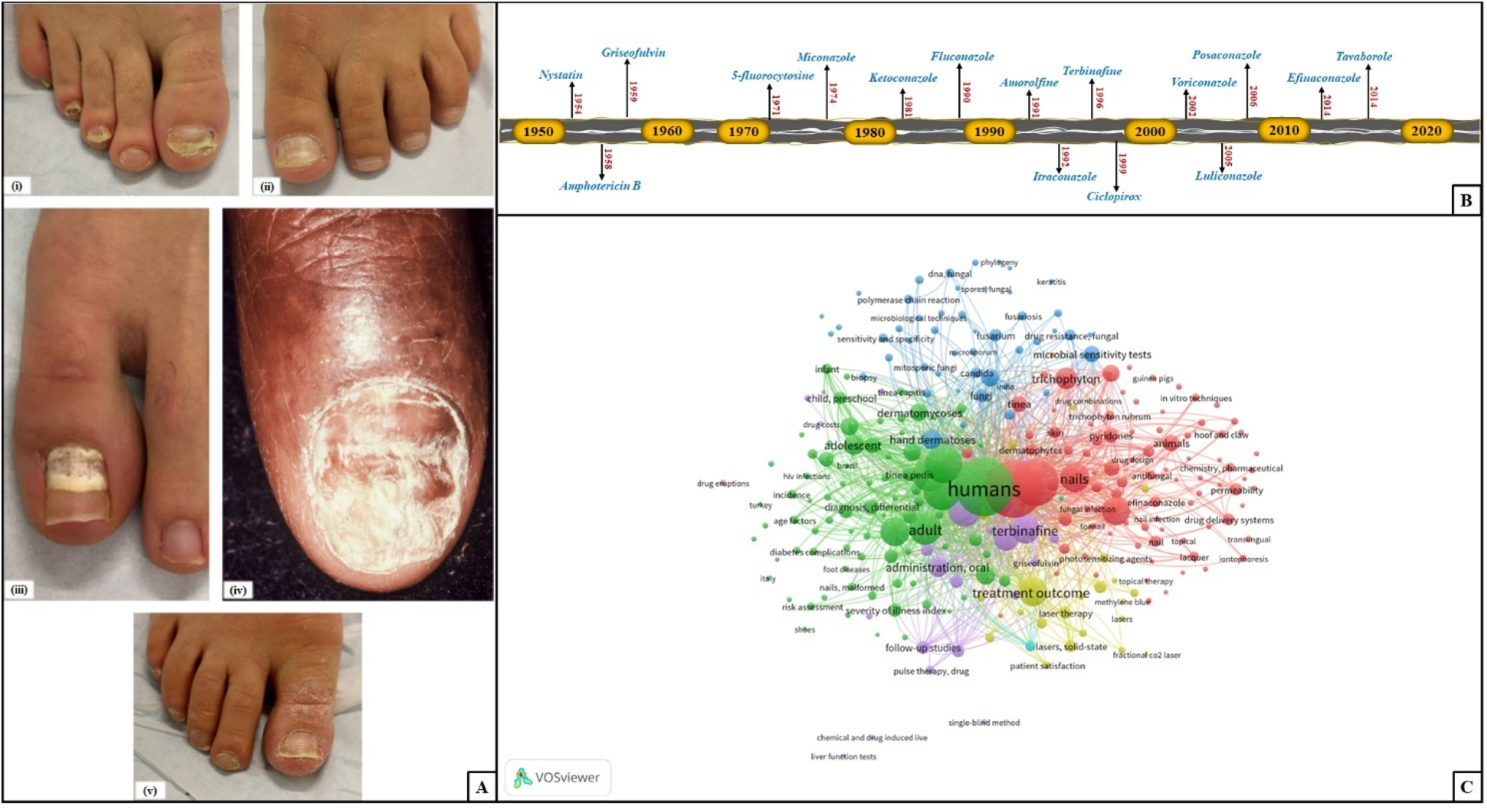
(A) Clinical subtypes of toenail fungal infection (this figure has been reproduced from ref. Lipner and Scher^19^ with permission from Elsevier, copyright, 2019). (B) Milestones in antifungal drug development for onychomycosis; (C) co-occurrence-based bibliometric analysis of available literature on the PUBMED database from 2000 to 2024 using the VOSviewer platform.

**Scheme 1:**
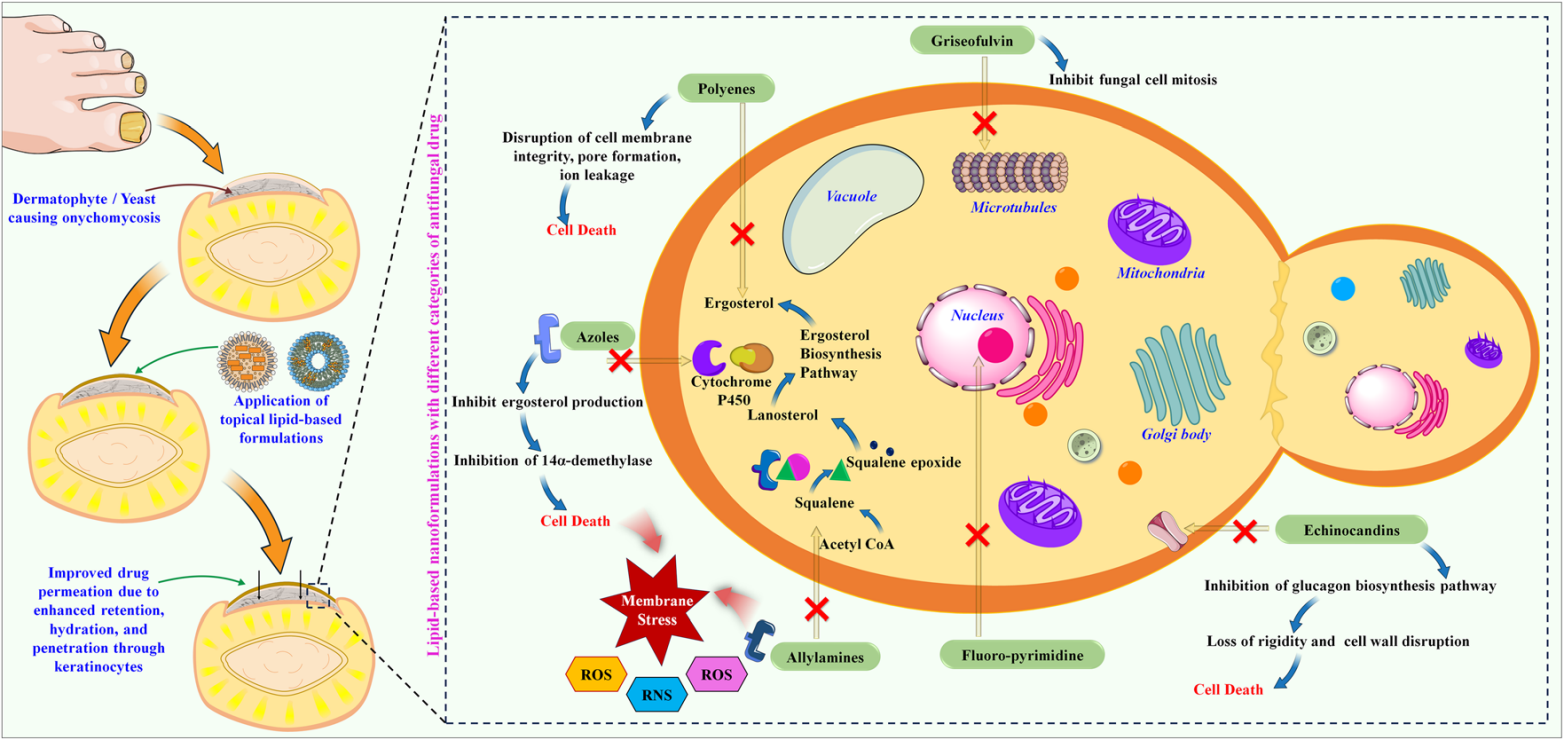
Schematic illustration of fungal nail growth and inhibition causative organism by ungual administration of lipid-based formulations.

Onychomycosis is characterized by fungal colonization within the nail bed and manifests with various clinical presentations depending on the causative fungal species, disease severity, and individual factors. The therapeutic management of onychomycosis presents numerous challenges due to the complex nature of the ailment and the unique composition of the afflicted nail structure.^1,2^ The nail serves as a challenging barrier, impeding the effective diffusion of topical antifungal substances to the underlying infection site, thus compromising treatment efficacy.^3^ Furthermore, the chronic and refractory characteristics of the condition, sluggish growth of nails, and presence of fungal spores in the environment may cause non-compliance by patients, necessitating prolonged therapeutic interventions.^4–6^ This challenge is compounded by the diverse fungal species responsible for onychomycosis, each with distinct sensitivity to antifungal agents.^7,8^ Correct identification of the causative agent is critical to deciding on appropriate therapy. Onychomycosis has various clinical subtypes (Fig. 1A). Distal Lateral Subungual Onychomycosis (DSO), caused by *T. rubrum* and *T. mentagrophytes*, begins at hyponychium and invades the nail as it progresses, causing discoloration and thickening.^9,10^ White Superficial Onychomycosis (WSO), mainly from *T. mentagrophytes*, affects the upper nail plate, creating white patches and making the nail rough and crumbly.^11^ Proximal Subungual Onychomycosis (PSO), often seen in immunocompromised patients, begins at the proximal nail fold with *T. rubrum* or *Fusarium* species, leading to leukonychia.^12,13^ Endonyx onychomycosis involves only the nail plate, caused by *Trichophyton soudanense* and *Trichophyton violaceum*, causing milky patches without nail bed involvement.^14,15^ Total dystrophic onychomycosis (TDO) signifies the culminating phase of any subtype, leading to severe nail destruction.^16,17^ The prevalence of onychomycosis varies significantly among various parts of the world. For the United States, it has been estimated to be between 2 and 14%. European rates are much higher, at approximately 23% of the population affected. For example, the United Kingdom has about 2.7%, while Germany has between 15 and 20%. It is more prevalent in France, between 20% and 30%. Its incidence is substantially high in the Asia-Pacific region; about 10% of the population of Japan translates to approximately 11 million people. In Latin America, the prevalence is relatively significant, especially with the high prevalence of diabetes and HIV infections. Both infections heighten the risk of acquiring the infection with these fungi. In the Middle East and Africa, onychomycosis is increasingly on the rise due to the sharp increase in diabetes. Countries in the region, such as Saudi Arabia, are dominated by enormous fungal infections that affect most individuals diagnosed with diabetes.^18^ The development of drug-resistant strains has complicated treatment strategies. A patient's compliance with a topical or systemic regimen often becomes an ongoing challenge, at least in regular application or administration. This convergence of multifactorial challenges makes the issue of onychomycosis not only a diffuse concern but also one that is complex in nature, such that the treatment of onychomycosis involves a complete, patient-specific approach that encompasses not only clinical resolution but also aesthetic improvement considering the individual's health. Only a few classes of antifungals are currently available to treat toenail infections; Fig. 1B shows a timeline of their discovery and market launch. Scheme 1 shows the mechanism of various antifungal drugs and targeted delivery by lipid-based formulations to treat fungal infections in the nail bed.

There are conventional approaches, such as systemic and topical treatments, and physical treatments, such as laser therapy. However, these approaches often have disadvantages, such as poor drug penetration, long treatment times, and increased relapse rates, highlighting the urgent need for innovative solutions. Lipid-based nanocarriers have been developed as a cutting-edge delivery method to circumvent the limitations of traditional antifungal formulations in onychomycosis. A compact keratinized nail plate is an impermeable barrier, which limits the penetration of the drug and prevents the effectiveness of local therapy. At the same time, antifungals administered systemically also lose effectiveness due to reduced bioavailability and systemic toxicity.^20^ These nanocarriers, including SLNs, NLCs, liposomes nanoemulsions, *etc.*, are molecularly optimized solutions for increasing solubility, stability, permeability, and drug retention.^20^ The lipid composition of these carriers is biocompatible. It interacts with the nail matrix through hydrophobic and van der Waal interactions, allowing drug permeation at deeper sites and long-term treatment. In addition, their ability to regulate the release pathways prevents the drug's initial degradation and systemic clearance, leading to prolonged drug release around the infection. Lipid nanotechnology as an antifungal treatment option has transformed the paradigms of drug delivery and provides precision-driven and targeted drug transport while circumventing the limitations of pharmacokinetics and drug resistance mechanisms. In addition to improved intracellular drug retention, these carriers, together with a lower dose regimen, prevent systemic toxicity improve therapeutic efficacy and patient compliance.^21,22^ Unlike conventional antifungals that act solely on fungal cells, lipid-based carriers can modify the nail structure, increasing hydration and softening the keratin barrier, thereby improving drug uptake and retention. By addressing the key problems of conventional antifungal treatments, lipid-based carriers propose a promising platform for the most effective treatment of onychomycosis and merit further translational and clinical research.

This review explores the pathophysiology and clinical attributes of onychomycosis, the status of available treatment, and discusses the challenges to effective treatment. Further, the review specifically focused on the advances and applications of lipid-based nanocarrier systems for treating onychomycosis, their ability to improve drug delivery, and achieving optimal therapeutic effects. These novelties are considered promising avenues to formulate more efficient and patient-oriented treatment approaches for onychomycosis.

## 2. Bibliometric analysis

VOSviewer, a popular software tool for generating bibliometric network maps and visualization, was used as the bibliometric analysis platform. Fig. 1C presents a thorough, co-occurrence-based bibliometric network analysis of terms related to onychomycosis or fungal infections of the toenails from the available literature in the PUBMED database from 2000 to 2024. It also provides a detailed account of the relationships between the clinical, diagnostic, and therapeutic areas within the onychomycosis research landscape. Key elements include “human,” “foot dermatoses,” “physical treatment approach,” “new treatment approach,” “transungual/topical administration,” and “treatment outcome,” with the links representing co-existing relationships between topics such as drug delivery systems, diagnostic techniques, and antifungal treatments. Also, prevalent research pathways and critical connections are highlighted that promote innovation and future directions in onychomycosis research and treatment (Fig. 1C).

## 3. Nail structure and clinical manifestations of onychomycosis

Anatomically, the nail includes the proximal nail fold (PNF), nail matrix, nail bed, and hyponychium, collectively making up the nail plate.^23^ The ungueal plate, *i.e.*, nail plate, is characterized by a flat rectangular shape with a translucent appearance composed primarily of keratin protein.^24^ Its flat, adherent, interlocking cells are densely packed and oriented perpendicular to the nail plate.^25^ The keratogenous zone of the nail bed that extends beyond the PNF is the whitish lunula, seen clinically and proximally, and covers about 1/4th of the total nail length.^26^ The matrix of the nail is substantially proliferative. It produces a nail plate and consists of three bonded layers.^26,27^

The nail plate primarily consists of hard α-keratin, with the dorsal layer potentially acting as a barrier to permeation.^28,29^ The nail bed is a pink, non-keratinized epithelium that connects the matrix to the hyponychium and contains living cells in the nail plate. Granular layers and sebaceous glands are absent.^30^ The hyponychium lies beneath the free distal end of the nail and has a protective function comparable to that of the lateral folds, the PNF, and the cuticle (Fig. 1).^31^ Drug delivery through the transungual route is essential for treating nail diseases, such as onychomycosis, psoriasis, paronychia, and onycholysis. Topical drug delivery is preferred to avoid systemic side effects and to provide direct benefits. However, a significant challenge is the poor permeability of drugs through the nail plate, which is attributed to the dense network of cross-linked keratin proteins.^32^ The human nail contains 2–35% water and 0.1–1% lipids, resembling a hydrophilic gel membrane with a lipophilic route, 10.60% disulfide linkage with 50–1000 μ thickness, 25% Maximum swelling capacity and 1.94 mg cm^−2^ h^−1^ water loss rate.^33^ Variations in solute penetration are due to nail properties. Traditionally seen as a fiber matrix, nails may have residual lipids, lowering the permeability to specific solutes. Cellular membrane remnants may partially obstruct the transport of hydrophilic solutes.^32,34,35^ Topical drugs are typically favored because of their localized action, without any side effects, which helps patients tolerate the therapy inexpensively.

The following are the sophisticated clinical presentation of onychomycosis: (i) dystrophic alterations mark the early stages, encompassing nail plate discoloration, often displaying hues of yellow, white, brown, or even a dark greenish-black tint as the disease advances. (ii) Progressive infection leads to hypertrophy and malformation of the affected nail. Altered contouring, including convexity or irregular shaping, emerges, potentially causing discomfort during footwear use. (iii) Nail fragility becomes evident, resulting in onychorrhexis, in which nails are susceptible to splitting or breaking, contributing to further structural compromise. (iv) The nail texture undergoes a friable transformation and is prone to crumbling into minute fragments, indicative of advanced pathology. (v) Progressive fungal growth induces onycholysis, which causes nail separation from the underlying nail bed. This separation causes discomfort and creates an interstice susceptible to secondary infections. (vi) Subungual hyperkeratosis signifies keratin debris accumulation beneath the nail plate. This accumulation resulted in nail elevation and interstitial spacing. (vii) In advanced instances, fungal degradation products contribute to the malodor emanating from the infected nail site. (viii) As the infection escalates, nail hypertrophy and malformation provoke discomfort and pain, accentuated during ambulation or footwear application. (ix) The contiguous spread of onychomycosis from one nail to adjacent ones is characteristic, resulting in progressive poly dystrophy, affecting multiple nails. (x) Impaired nail defense mechanisms render nails susceptible to secondary pyogenic bacterial infections. Ensuing bacterial colonization accentuates inflammation and exacerbates patient discomfort.

## 4. Pathogenesis of fungal nail invasion

**Fig. 2:**
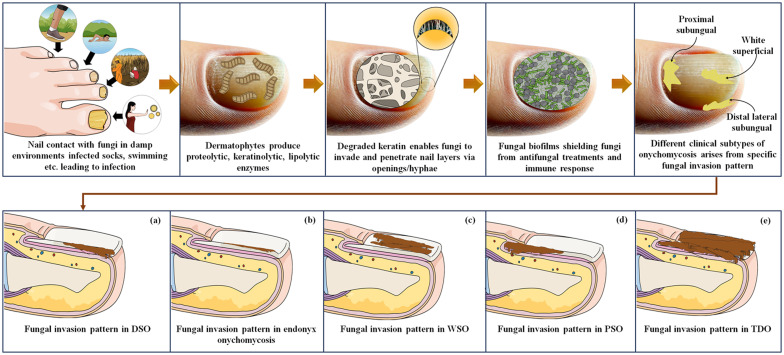
Pathogenesis for fungal nail invasion represents keratin degradation, biofilm formation, and invasive patterns that cause different clinical subtypes of onychomycosis: (a) DSO; (b) endonyx onychomycosis; (c) WSO; (d) PSO; (e) TDO.

Onychomycosis usually begins upon contact of the nail with an agent of fungi such as dermatophytes, non-dermatophyte molds, and yeasts in settings that promote the growth of fungi. Following this specific contact, the fungus secretes particular enzymes capable of degrading keratin, allowing it to penetrate deeper into the nail. The formation of biofilms leads to a reduced immune response in the area of the nail processes, which aggravates the infection (Fig. 2). Thus, it becomes difficult to manage therapeutically. Onychomycosis is transmitted through direct contact between the nail and dermatophytes, non-dermatophyte molds, or yeasts.^36^ This contact can occur in various settings, such as damp environments, locker rooms, or swimming pools, where fungi thrive. When nails encounter these fungal agents, they can adhere to the nail surface and initiate infection. Fungi, especially dermatophytes, produce proteolytic, keratinolytic, and lipolytic activity enzymes. Proteolytic enzymes break down proteins, keratinolytic enzymes degrade keratin, and lipolytic enzymes digest lipids.^36,37^ In onychomycosis, these enzymes work together to degrade the tough keratin in the nail plate, enabling the fungi to access nutrients within the nail. This enzymatic action softens nail structure, allowing fungal penetration.^38^ Once keratin is degraded, fungi can easily invade the nail.^39^ They penetrate through small openings, injuries, or separations between the nail plate and bed. Softened keratin provides a pathway for fungal hyphae (thread-like structures) to infiltrate the nail layers, starting from the hyponychium.^40–42^ This reduced immune activity makes nails susceptible to fungal invasion. Unlike the skin or mucous membranes, immune cell access to the nail plate is restricted, allowing fungi to establish infections with less resistance.^43,44^

Fungal biofilms are microorganisms surrounded by a protective layer of proteins, sugars, and other substances.^45^ These biofilms form within the nails as a defense mechanism, making it difficult for antifungal agents and immune cells to reach and eliminate the infection. The biofilm creates a barrier that protects the fungi from the immune system and makes the treatment less effective.^46,47^ Clinical subtypes of onychomycosis can arise depending on the location and manner of the invasion within the nail. For instance, the invasion that starts in the hyponychium and moves distally is known as the distal lateral subungual subtype. The white superficial subtype comprises invasion within the upper nail plate surface. The proximal subungual subtype extends distally from the proximal nail fold. The unique invasion pattern of each subtype gives rise to its characteristic clinical features (Fig. 2).

## 5. Conventional antifungal therapies and associated challenges with transungual drug delivery

The main reason for the challenge in managing onychomycosis is the not-so-understood intrinsic pathophysiological mechanisms, and other factors that complicate the scenario include characteristics of fungal growth and particular susceptibility of the individual patient. Various approaches to improving drug delivery to the nail to treat nail infections and disorders effectively have already been developed. In any case, it should be noted that every method of pulsed lasers and ultrasound-mediated delivery to traditional topical and oral therapies has its specific mechanism, advantages, and challenges. This comparative analysis facilitates clinical practitioners in making well-informed decisions by suggesting a detailed understanding of the benefits and shortcomings of each technique. Each technique is discussed in Table 1.

**Table 1:** Different approaches for onychomycosis treatment with their key insights

Approach	Mechanism	Key considerations	Benefits	Challenges	Ref.
Pulsed lasers	Pulsed laser disrupts the integrity of keratin chains in the nail, creating craters or holes to allow for deeper drug penetration	Laser intensity and duration: choice between removing the nail or creating perforations	Allows direct drug application to the site of infection; reduces relapse rates, especially with carbon dioxide lasers in onychomycosis	Risks include thermal damage and uncertain outcomes in some cases	29, 48 and 49
Ultrasound mediated delivery	Low-intensity ultrasound is used to increase permeability by cavitation or formation of pits	Acoustic power and exposure duration	It enhances drug flux threefold, reduces treatment time, and minimizes side effects associated with systemic therapy	Technology needs more testing for broader use	50 and 51
Iontophoresis	It uses an electric field to enhance transungual permeation by electrophoresis and electroosmosis	pH, buffer ionic strength, current density (maximum 0.5 mA cm^−2^)	Achieves higher drug concentrations within a shorter time, making it practical for fungal infections	It requires a controlled electric field and limited penetration of larger molecules	52–55
Hydration	Water acts as a plasticizer, increasing pore size and facilitating transungual permeation	Humidity level, water loss, ceramide concentration, and water-binding capacity	Increased permeation threefold with higher relative humidity (100%) improves nail condition in diseases like onychomycosis	Prolonged exposure to water may weaken nails; efficacy depends on hydration status	41 and 56
Etching	It uses chemicals like phosphoric acid to create microporosities that increase surface roughness, improve drug penetration and bonding, promote the reduction of contact angle, enhance wettability	Roughness, wettability, and interpenetration of the delivery system	Enhances permeability and interpenetration of antifungals	It can lead to surface damage if overused and limited to localized treatment	48, 57 and 58
Topical applications	Involves direct application of antifungal or therapeutic agents over the nail plate for localized drug delivery	Drug formulation, penetration enhancers, frequency of application	Minimizes systemic side effects and allows direct treatment of fungal or bacterial infections	Often limited by poor permeability of the nail plate, leading to insufficient drug concentrations at the target site	59–61
Oral therapy	Involves systemic absorption and subsequent transport of drugs through the bloodstream to the affected nail tissue	Absorption rate, metabolism, bioavailability, and potential for drug interactions	Provides a comprehensive treatment for infections, including those affecting the nail bed	A higher risk of side effects, including liver toxicity and gastrointestinal disturbances, often requires prolonged treatment	62 and 63

### 5.1 Challenges associated with transungual drug delivery

The treatment modalities for onychomycosis face several challenges that require developing novel therapeutic approaches.^64^ There are several challenges in effectively treating onychomycosis, a fungal infection of the nails. These challenges include the rigid structure of the nail, which makes it difficult for medications to penetrate, the high cost of treatment, the prolonged duration of therapy required, and the high probability of disease recurrence. These factors contribute to the barriers to successful outcomes in treating onychomycosis.^65^ Oral antifungals have a limited safety profile, often resulting in concerns regarding liver toxicity and drug interactions. Topically applied antifungal agents can penetrate the nail plate *in vitro*, but their penetration is insufficient to penetrate the deeper keratinized layers and matrix. This limitation limits its use to superficial infection only, but clinical studies are still inconclusive. This more extended presence of the active ingredient in the nail enables weekly dosing, a shortened treatment period, or pulse treatments. Combining topical and oral antifungal agents acting on the nail from multiple sides is an optimal pharmacokinetic approach. The high porosity, water-resistant, dense nail plate matrix is such a core limitation of drug penetration that it confines the efficacy of these agents against any deeper-lying infections. This poor penetration limits topical therapies to superficial mycoses, which have limited chances of a successful outcome; therefore, the infection is only limited to the outer layers of the nail. Antifungal molecules can reach the nail bed and the nail matrix *via* the blood circulatory system with the oral/systemic route of administration. Because onychomycosis is challenging to treat, it often takes more than 18 months to achieve a complete cure, and 20–25% of cases do not respond to treatment. Doctors become aware of rapid recurrence because either fungal spores or hyphae remain. The slow growth rate of toenails, the thickness of the nail keratin, and the persistence of the fungus in the environment (*e.g.*, in shoes) influence its effectiveness. On the other hand, the immune response and the high keratin density in the closed nail bed are responsible for the complexity of complete healing.^66^ Onychomycosis usually persists for a long time, mainly if effective treatment is not provided. Biofilms, complex communities of microorganisms that adhere tightly to epithelial surfaces *via* an extracellular matrix, can be formed by fungi. The development of these biofilms represents a barrier to treatments, which is a significant factor in the high rate of recurrence, relapse, and treatment failure observed in onychomycosis. This defensive measure highlights the challenge of eliminating the infection and developing novel therapeutic approaches to combat biofilm resistance.^67,68^

#### 5.1.1 Limited nail penetration

Penetration of the drug into the nail matrix is often insufficient, limiting the effectiveness of treatment. This is partly caused by the thickness of the keratin in the nails, the slow growth rate of toenails, and the ability of fungi to survive in conditions such as shoes. Because of these factors, it is difficult to eradicate fungal infections with topical treatments. The rugged, protective nail plate, the storage of pathogens between the plate and the nail bed, and the slow growth of the nail also cause problems. The barrier properties of the nail plate make it even more challenging to deliver antifungal drugs to the tissue in the concentrations required to kill fungi deeply embedded in the nail bed. When comparing drug penetration, weight-based measurements or highly concentrated solutions or suspensions have generally been used.^69^

#### 5.1.2 Fungal resistance mechanisms

Antifungal resistance has significant implications for treating onychomycosis and other fungal infections. Resistance can lead to reduced treatment effectiveness, and problems such as low adherence and long treatment durations can further promote the development of resistance. Non-compliance with treatment can lead to frequent relapses, leading to the selection of resistant fungal strains.^70^ Longer disease duration and decreased antifungal efficacy allow the fungi that cause onychomycosis to spread and become more invasive. Antifungal resistance can be inherent. That is, it arises without prior drug exposure or is acquired using antifungal drugs.^71,72^ Microbiological resistance mechanisms include gene mutations or changes in gene expression, which can reduce drug absorption, induce structural changes, accelerate drug degradation, or increase drug efflux. Biofilms also play a role in mediating resistance by protecting the fungi from the immune system and drugs.^73,74^ Despite proper medication intake, clinical resistance persists, *i.e.*, the interaction between patient, drug, and fungus. Correct diagnosis and ongoing treatment are among the basic measures for resistance prevention and treatment of onychomycosis.^5^

#### 5.1.3 Host immunity variability and impact of comorbidities

Variabilities in the host immune response, such as those seen in immunocompromised individuals, can impact treatment outcomes and lead to chronic or recurrent infections. Nails pose a significant challenge for treatment due to their lack of intrinsic immune function and impenetrability. Factors such as a weakened immune system (standard in HIV patients), diabetes, and peripheral vascular disease can affect treatment success. ^75–77^ In addition, conditions such as tinea pedis, age, and obesity increase the risk, while comorbidities such as diabetes and psoriasis increase susceptibility to disease. Effective treatment is often hampered by slow nail growth, patient comorbidities, and the reluctance of prescribers and patients to take oral medications due to perceived toxicity concerns. The threat of developing onychomycosis is significantly increased by several underlying medical conditions, including nail trauma, nail psoriasis, tinea pedis (athlete's foot), and patient characteristics such as age and obesity. Comorbid medical conditions that increase susceptibility to this fungal infection include diabetes mellitus,^78^ various cancers,^79^ immunodeficiency disorders,^80^ and peripheral arterial disease.^81^ In addition, a genetic predisposition to onychomycosis has been identified, suggesting that certain people may be predisposed to the condition. A study conducted at Buea and Limbe Regional Hospitals found a prevalence of onychomycosis to be 50.7% in 152 diabetic patients. *T. rubrum* was the most common fungus (62%), followed by *T. metagraphyte* (22%) and *T. tonsurans* (16%). No significant sociodemographic or clinical predictors were identified. The fungi were most sensitive to miconazole (66%), with less sensitivity to amphotericin B (19%) and ketoconazole (14%).^82^ In a study of 38 patients with psoriasis and/or psoriatic arthritis, 57.89% were diagnosed with onychomycosis. Positive results were detected by direct mycological examination in 44.8%, culture in 42.1%, and histopathological examination in 31.6%. *T. rubrum* was the most common dermatophyte, and yeasts such as *C. parapsilosis* and *C. albicans* were also present. The study found a high rate of onychomycosis (92.8%) in patients taking methotrexate alone, suggesting a possible association between psoriatic disease, immunosuppressive therapy, and fungal infections.^83^

#### 5.1.4 Safety profiles and side effects

Prolonged use of oral antifungals can lead to liver toxicity and drug interactions, especially in vulnerable groups such as the elderly and diabetics. Azole antifungals are responsible for serious drug reactions, including QT prolongation, torsade de pointes, heart failure, rhabdomyolysis, and even death. Topical antifungals used to treat onychomycosis can also cause local side effects, with efinaconazole potentially triggering allergic skin reactions.^84^ Both patients with and without a history of liver disease may experience increased liver enzymes after taking terbinafine.^85^ While terbinafine rarely causes side effects in patients with onychomycosis, those who suffer from onychomycosis often report changes in taste and smell. Although it is unclear exactly how terbinafine causes taste disorders, it is thought that taste receptor dysfunction may be due to the inhibition of cytochrome P-450-dependent enzymes.^86^

#### 5.1.5 Environmental re-exposure and reinfection

The risk of recurrent onychomycosis is strongly influenced by environmental factors, including inappropriate footwear and shared public spaces. Repeated contact with contaminated textiles, shoes, and socks can lead to a new fungal infection. It is known that dermatophytes can colonize in shoes and remain there for long periods by feeding on trapped skin cells and sweat.^73,87^ In addition, blankets, sheets, and towels can serve as harbors for fungi. After antifungal treatment, wearing contaminated socks and shoes can facilitate reinfection. In addition, the risk of reinfection may be increased if textiles and shoes are stored together or washed in the same machine, as cross-contamination of sterile laundry can occur.^88^

#### 5.1.6 Aging and nail growth impairment

Treating and monitoring onychomycosis in older people is problematic because nails grow more slowly and take longer to show signs of improvement. Since recurrences are common, older people should develop good hygiene practices and change their lifestyles accordingly. In the long term, topical antifungals one to three times per week for prevention can reduce recurrences. In addition, it is extremely successful in treating the primary infection and keeping the nail structure firm and in good condition.^89^ Amorolfine and ciclopirox are lacquer-based topical antifungal agents that pose a practical challenge for elderly patients due to frequency of use, effective nail removal, and prolonged treatment duration. However, this was not as practical for these older people as they could not carry out demanding protocols. Therefore, options for treating onychomycosis in this age group with a more straightforward application and maintenance schedule might be worthwhile.^90^ some nail characteristics make the treatment ineffective. These are onychogryphosis and onycholysis, slow nail growth, and greater nail matrix or plate involvement, which affect over 75 percent. These factors complicate treatment and reduce its effectiveness.^91^ In addition to the high prevalence of peripheral vascular disease, diabetes, immunosuppression, and physical trauma in the elderly, which are also responsible for poor treatment response or recurrence, there are other factors of interest. On the other hand, patients' lack of compliance, poor hygiene, and unsuitable footwear are mainly responsible for this. These are the critical points that should be addressed to improve the result.^92^

#### 5.1.7 Cost and accessibility of advanced treatments

Main patient compliance and clinical outcome appear to be significantly compromised by costly treatment options for onychomycosis. In certain cases, the financial burden may result in inadequate or incomplete treatment of a disease, promoting the growth of numerous pathogens and increasing the risk of recurrence. Non-confirmation burdens healthcare systems as it leads to poor health and increased resistance to fungi. Therefore, accessible and affordable treatment options are required for effective treatment, and timely education about the importance and cost of treatments is necessary to increase patient adherence. According to Yousefian and colleagues, the cost of treating onychomycosis varies greatly depending on the treatment plan. Unlike oral therapies, which typically cost $100 or less per month, topical therapies can cost between $100 and $500. PDT, LLLT, and laser treatments can cost between $100 and $1500 per month for a maximum of two months. The average monthly cost for OTC medications and plasma therapy is between $100 and $500.^93^ When treating onychomycosis, it is critical to consider the patient's financial situation and insurance coverage due to the chronic nature of the disease and the possibility of recurrence.

### 5.2 Challenges specific to lipid-based nanoformulation

#### 5.2.1 Stability

Lipid-based nanomaterials, such as liposomes, SLNs, and NLCs contend with stability challenges due to particle aggregation, lipid phase transitions, and drug leakage.^94,95^ Lipid composition, surfactant selection, storage conditions, and pH or ionic strength influence stability. Strategies to enhance stability include the incorporation of cryoprotectants (*e.g.*, sucrose, trehalose) during freeze-drying, PEGylation to improve colloidal stability, optimization of lipid ratios, and adjustment of zeta potential to prevent aggregation. Liposomes are prone to drug leakage, while SLNs may undergo drug expulsion as a result of lipid recrystallization. In contrast, NLCs offer improved stability due to their imperfect lipid matrix. Maintaining stability necessitates careful formulation design, proper storage at low temperatures, and advanced stabilization techniques such as lyophilization.^96,97^

#### 5.2.2 Scalability

Scaling up the manufacturing of lipid-based nanomaterials presents several challenges, including maintaining consistent particle size distribution, managing complex multi-step processes, ensuring sterility, and addressing batch-to-batch variability. Key issues include particle size control, thoroughly mixing lipid components and active ingredients, equipment limitations, and solvent removal.^98^ Ensuring sterility, robust process monitoring, and formulation optimization are also crucial. Potential solutions include using microfluidic technology, high-pressure homogenization, single-use systems, and advanced characterization techniques. Collaboration with experienced formulation scientists and process engineers is essential to address these challenges effectively.^99,100^

#### 5.2.3 Toxicity concerns

Lipid-based nanomaterials hold promise for drug delivery but present notable toxicity concerns that must be carefully addressed. The composition of lipids is a critical factor, as certain lipids can trigger immune responses or induce toxicity. At the same time, oxidation and phase transitions may generate reactive oxygen species, leading to cellular damage.^101,102^ Particle size and surface charge influence biodistribution, with aggregation potentially causing organ accumulation, particularly in the liver and spleen, increasing toxicity risks.^103^ Additionally, interactions with plasma proteins can lead to protein corona formation, altering targeting efficiency and immune recognition.^104,105^ Immune responses, including inflammation and antibody development upon repeated administration, may further limit their clinical application.^106,107^

## 6. Designing of lipid-based carrier for transungual drug delivery

**Fig. 3:**
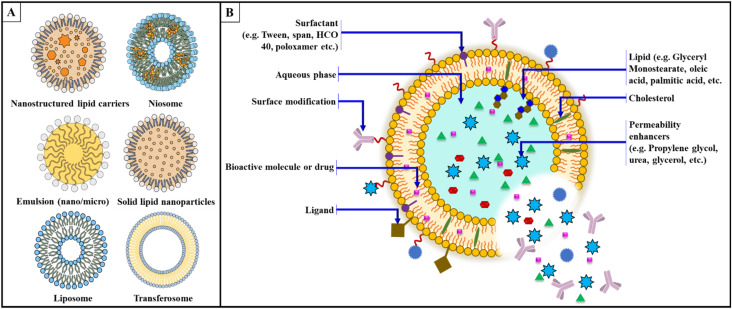
(A) Different types of lipid-based delivery vehicles; (B) designing of lipid-based drug delivery system.

Designing a lipid-based formulation for transungual drug delivery presents challenges due to the dense keratinized structure, which significantly hinders drug permeation. This keratin matrix further restricts the diffusion of therapeutic agents by restricting lipid fluidity and hydration and acting as a physical barrier. To overcome this natural barrier, transungual formulations must be specifically developed. In the case of API, as the molecule size increases, drug penetration would decrease. Conversely, the penetration of the active ingredient decreases as the molecular size increases. Except for miconazole, which penetrates the nail plate regardless of pH, it should be noted that different antifungal drugs have different acid strengths. In addition, efinaconazole and tavaborole do not bind to keratin. The permeability coefficient initially decreases as the lipophilicity of the diffusing alcohol molecule increases. However, an additional increase in lipophilicity leads to a higher permeation rate once a particular threshold value is exceeded.^108^ Nail permeability was studied in terms of the drug's molecular weight, ionic nature, and lipophilicity. Permeability was shown to be unaffected by lipophilicity, but it was considerably lower for ionic drugs and decreased as molecular weight increased. According to a model based on these two variables, permeability is independent of the octanol/water partition coefficient but decreases with increasing molecular weight. Ion hydration increases the apparent molecular weight, which is likely why the dissociation of the drug further reduces permeability. Healthy nail plates and nail plates with fungal infection appear to have similar permeabilities.^109^ Keratins, the primary structural proteins in human nails, have an isoelectric point (pI) between 4.0 and 5.0. These keratins have a net positive charge at pH values below their pI and a net negative charge above their pI. The surface of the nail plate has an apparent pH of about 5.0, while the interior has an approximate pH of 4.1. Under normal physiological conditions, the nail plate is approximately neutral.^35,110^ The weakly acidic or basic ionization of API is modulated by the pH of aqueous formulations, altering the hydrophilicity or lipophilicity of these active ingredients. These manipulations directly and significantly affect the solubility of the materials in the formulation matrix, their solubility in the keratinized nail plate, and their interactions with their keratin matrix.^111,112^ The susceptibility of fungi to certain antifungals is influenced by pH. In particular, naftifine's *in vitro* antifungal activity against *T. mentagrophytes* decreases as the pH decreases from 7 to 4.^113^ Lipids are considered an essential component of topical drug delivery systems intended to act as systemic therapeutics across the skin. These improve the penetration/retention of the drug in the skin. Two primary lipid excipients commonly used in these formulations are fatty acids, triglycerides, phospholipids, and sterols such as cholesterol. They support the formation of efficient and malleable vesicles, the solubility of drugs, and the permeability of the skin barrier.^114^ Surfactants are added to the formulation to adjust the release profile and improve drug delivery. Surfactants with an HLB greater than or equal to 12 reduce the interfacial tension at the interface, which aids in formulating stabilized colloidal dispersions or vesicular carriers.^115^ Hydrolysed vegetable oils and polyethylene glycol are familiar sources of these materials, including ethoxylated alcohols and polysorbates. Polyoxyl-hydrogenated castor oil (Cremophor) is widely used for this purpose.^116^ Surfactants increasing drug solubility aggravate the bioavailability while also controlling the drug release characteristics and improving pharmacokinetic parameters, possibly by inhibiting efflux transporters (Fig. 3) (Table 2).^132^

**Table 2:** Examples of lipid-based excipients for transungual drug delivery

Chemical name	Synonym	Melting point (°C)	Functionalities	Ref.
Glycerol behenate	Compritol 888 ATO	65–77	Viscosity enhancer, sustained-release, lipidic coating excipient	117
Glyceryl caprylate	Capmul	30–39	Moisturizer, preservative, permeability enhancer, emulsion stabilizer, solubilizer	118
Glyceryl monostearate	Capmul GMS-50	55–60	Lubricant, emulsifier, sustained-release ingredient, emollient, stabilizing agent	117
Glyceryl tristearate	Tristearin	72–75	Controlled release, thickening agent	119
Glyceryl palmitostearate	Precirol ATO 5	52–55	Sustained-release agent	120 and 121
Hydrogenated castor wax	Castorwax, Kolliwa® HCO	85–88	Provide stiffness, thickener, emollient	122 and 123
Cholesterol	—	147–150	Emollient, emulsifying agent, improves rigidity and stability	124
Stearic acid	Cetylacetic acid, Kortacid 1895	>54	Emulsifying agent, solubilizing agent	125 and 126
Palmitic acid	Cetylic acid, Emersol 140	63–64	Emulsifying agent, penetration enhancer	127
Oleic acid	Crodolene	13–14	Emulsifying agent, penetration enhancer	128 and 129
Medium-chain triglycerides	Miglyol 812		Emulsifying agent, solvent	129
Castor oil	EmCon CO; Lipovol CO	−12	Stabilize the emulsion, emollient, oleaginous vehicle, solvent	130
Linoleic acid	Emersol 310	−5	Emulsifying agent, penetration enhancer	131

## 7. Leveraging lipid-based formulations for effective onychomycosis treatment

Novel approaches enhance topical antifungal therapy, addressing conventional treatment limits and the high relapse rate of onychomycosis. Nanotechnology-based approaches modulate drug stability, release kinetics, and residence time, transforming onychomycosis treatment. Target-specific drug delivery systems for administering pharmaceutical compounds enable drug localization to the diseased sites. These novelties support antifungal drug penetration using lipid-based formulations, NEs, MEs and heightened delivery depth. Targeted drug delivery *via* liposomes, NLCs, and nanoparticles directly direct antifungal agents to the infection site, enhancing efficacy, minimizing side effects, and optimizing outcomes. Sustained drug release is fundamental, allowing controlled and extended delivery, reducing frequent applications, improving convenience, and maintaining therapeutic levels for combatting persistent infection. These systems mitigate systemic exposure by delivering drugs directly to the infection site, reducing the risk of systemic side effects and thus enhancing safety.

Lipid-based formulations provide several advantages over conventional antifungal treatments, such as significantly enhancing hydrophobic antifungal drugs' solubility, stability, and transungual penetration by encapsulating the latter in amphiphilic lipids. Due to their nano size, lipid nanocarriers enable deep diffusion of drugs through microchannels in the nail plate, unlike traditional topical formulations that are unable to penetrate the compact keratinized nail matrix. With a solid lipid core, SLNs inhibit burst release and ensure persistent therapeutic activity by regulating drug release *via* matrix degradation and diffusion processes. Because of this prolonged drug retention, fewer doses are required, a major drawback of traditional creams requiring multiple daily dosages. Moreover, unlike conventional remedies, which are unstable, SLNs protect encapsulated antifungal drugs from oxidation and enzymatic degradation, extending their shelf life. The partly amorphous matrix of NLCs, a modified version of SLNs, provides better stability and a greater drug-loading capacity by combining liquid and solid lipids. This structural flexibility improves medication absorption by decreasing crystallinity and reducing drug expulsion. Liquid lipids, which are absent from traditional topicals, modulate lipid fluidity and enhance diffusion in NLCs, further improving penetration through the hard structure of the nail. Additionally, through hydrophobic and van der Waals forces, lipid-based systems have robust interactions with keratin, enhancing adhesion and extending drug retention at the infection site. Hydrophobic antifungals are well soluble in nanoemulsions composed of water, oil, and surfactants. This ensures that the antifungals are well distributed and penetrate the nail bed. Their exceptionally tiny droplet size (less than 100 nm) improves surface area and promotes the absorption of drugs in contrast to conventional emulsions or ointments, which have limited penetration and spreading. Compared to conventional topical treatments that mostly stay on the nail surface, adding surfactants in nanoemulsions increases drug dispersion by further aiding in the disruption of the dense keratin network. Additionally, lipid-based nanoformulations reduce systemic toxicity, a significant disadvantage of oral antifungal treatments, which frequently result in gastrointestinal adverse effects and liver damage because of the extensive drug distribution. While retaining high therapeutic concentrations at the infection site, SLNs, NLCs, and nanoemulsions minimize systemic exposure by facilitating localized drug delivery. Furthermore, by ensuring regulated and prolonged drug release, these systems lessen the need for frequent dosing that traditional formulations need. Their therapeutic effectiveness is further increased by their capacity to modify medication partitioning and retention at the molecular level.

### 7.1 Mechanism of lipid-based formulations penetrating the nail plate

Nanocarriers facilitate transungual delivery by leveraging their small size to cross the minute spaces interspersed among keratin fibers within the nail plate and periungual skin, releasing the drug into the nail bed.^34,133,134^ This enables the potential disruption of the tight junctions within the nail plate and may be further enhanced using penetration enhancers that disrupt the disulfide bonds in keratin, allowing for the deeper infiltration of encapsulated drug molecules. The nanoscale dimensions allow them to navigate inter-fiber gaps within the nail plate, which are otherwise inaccessible to larger molecular entities. Lipid-based nanocarrier formulations integrate penetration enhancers, such as thiols (*e.g.*, cysteine, glutathione) or sulfites, that facilitate the breakdown of disulfide bonds in keratin, thereby loosening the protein structure and promoting drug penetration.^65,135^ Additionally, some nanocarriers possess hygroscopic properties, enabling them to absorb moisture from the surrounding environment, resulting in slight hydration of the nail plate and subsequent enhancement of drug diffusion.^136,137^ Furthermore, the surface properties of nanocarriers can be meticulously engineered to interact favorably with the nail plate, thereby promoting adhesion and optimizing drug delivery. Ultimately, these advancements enable personalized medicine and tailoring treatment based on factors such as infection severity and medical history, thereby improving outcomes and satisfaction (Fig. 3).

### 7.2 Applications of different lipid-based formulation in onychomycosis

#### 7.2.1 Microemulsion

**Fig. 4:**
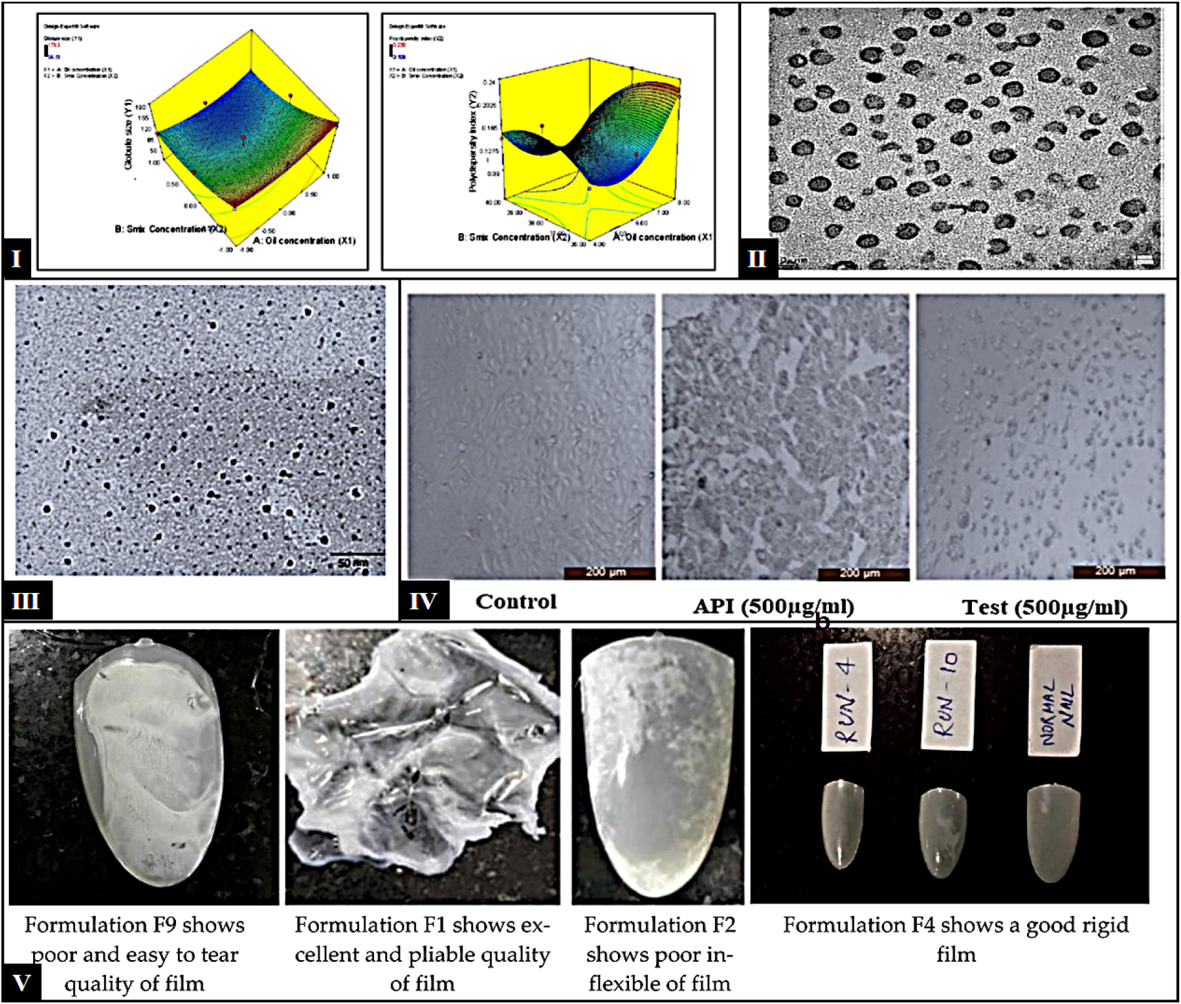
(I) 3D response–surface plots – globule size (left) and PDI (right); (II) TEM micrograph of TLM showing uniform and small size globules less than 50 nm (this figure has been reproduced from ref. Agrawal *et al.*^143^ with permission from Elsevier, copyright 2022); (III): TEM of developed efinaconazole MEs and (IV) cytotoxicity study of control, API, and test formulation (this figure has been reproduced from ref. Agrawal *et al.*^144^ with permission from Elsevier, copyright 2021). (V) TEM of Amorolfine HCl NEs(V) different films obtained during the optimization (the figure adapted from Fatima *et al.*^145^ under CC BY license).

MEs are thermodynamically stable transparent colloidal carriers with globule sizes ranging from 10 nm to 100 nm, composed of a lipid phase, aqueous phase, emulsifier, and co-emulsifier.^138,139^ MEs composition can facilitate the permeation of therapeutic agents across the skin and nails. The low viscosity of MEs is a significant challenge because of their minimal retention when applied topically. However, the retention of ME-based formulations can be improved by utilizing viscosity modifiers or gel formation.^140^ Barot and colleagues developed and optimized MEs using a d-optimal design. MEs were formed in the gel using a carbopol polymer. Permeation of the developed formulations was evaluated using human cadaver skin. The permeation of terbinafine from the MEs and MEs gels was about 4.2 and 2.7-fold higher, respectively, than those of the marketed formulation after 12 h. The higher permeation was attributed to the smaller globule size of MEs, which provides a larger surface area. The antifungal activity of the MEs gel also revealed promising results against *C. albicans* and *T. rubrum* compared to marketed formulations, confirming the potential of formulations for treating onychomycosis.^141^ Another study by Thatai and Sapra reported the delivery of terbinafine hydrochloride utilizing MEs and optimized it *via* a d-optimal design. Two different permeation enhancers, *N*-acetyl-l-cysteine (NAC) and urea, are used alone or in combination to improve drug permeation. The permeation study was performed on an animal hoof membrane, where the receptor medium was PBS (pH 5.8) containing 1% Tween 80. The MEs gel comprising both permeation enhancers showed improved drug retention, that is, ∼6.6-and ∼9.2 folds higher in the hoof than in the MEs and marketed formulations, respectively. This difference is attributed to the short residence time on the nail plate owing to the low viscosity of the MEs. At the same time, the oily base of the marketed cream formulation might have prevented the drug from being transported across the nail plate. The morphological characterization by SEM showed that untreated hooves showed smooth surfaces and with very few striations; water-treated hooves displayed swelled, and layer loosened; hooves treated with 5% urea or 5% NAC showed uneven surfaces with irregular pores, and pores even more numerous in the case of urea treatment and treated with a 1 : 1 mixture of urea. NAC showed increased disruption, bigger pore size, and more pronounced surface alterations, which suggested synergistic action between the two enhancers.^142^ Agrawal and colleagues developed and optimized a MEs formulation of TVB for the targeted treatment of onychomycosis, employing a 3^2^ factorial design (Fig. 4-I). The study demonstrated that the cumulative permeation of TVB was significantly enhanced in the TVB-loaded microemulsion (TLM), exhibiting 2.0 times greater permeation than the reference formulation and 1.4 times greater than the TLM gel. TEM photograph is presented in Fig. 4-II; the reduced globule size of TLM facilitates deeper penetration into the nail matrix, while its lamellar microstructure supports sustained drug release, highlighting its superior efficacy. Results from nail clipping studies aligned with *ex vivo* bovine hoof membrane permeation data, indicating that the TLM gel's elevated viscosity might hinder nail penetration and hydration. The investigation confirmed that TLM exhibited significantly enhanced antifungal activity compared to reference formulations, with approximately 2.67 times greater inhibition against *T. rubrum*, 2.72 times against *T. mentagrophytes*, and 1.64 times against *C. albicans*. Results from nail clipping studies aligned with *ex vivo* bovine hoof membrane permeation data, indicating that the TLM gel's elevated viscosity might hinder nail penetration and hydration. Additionally, the TLM gel displayed significant antifungal potency, with 2.23 times greater inhibition for *T. rubrum*, 2.30 times for *T. mentagrophytes*, and 1.48 times for *C. albicans*, confirming the potential of these formulations.^143^ Agarwal and coworkers developed efinaconazole-loaded MEs and optimized them using 3^2^ factorial designs, and their potential for transungual delivery was evaluated. In *ex vivo* permeability performed on the bovine hoof membrane, the permeability was significantly higher for both MEs and ME gel over 24 hours. This is considerably higher than the permeability measured for the reference drug formulation. The poor permeability of the free drug might be due to binding with nail keratin. The globules appeared dark in ME-based formulations due to the smaller globule size. Fig. 4-III showed a more significant concentration gradient in the nail-affected area. Antifungal activity of *T. rubrum*, *T. mentagrophytes*, and *C. albicans*. The ME formulations showed significantly better antifungal activity than the reference formulations. Moreover, cytotoxicity data reported no toxic effects of excipients evaluated in the Vero cell line compared to free API and control (Fig. 4-IV).^144^

#### 7.2.2 Nanoemulsions and nanoemulgel

NEs serve as carriers to deliver therapeutic ingredients, characterized by droplet sizes ranging from 20 to 200 nm. Their excellent thermodynamic and kinetic stability makes them an ideal choice over other carrier systems.^146–148^ Such type of carrier requires a minimum concentration to stabilize and effective topical drug delivery attributed to their nano size, considerable surface area, and minimum surface tension, helping them to penetrate rapidly with enhanced efficacy and tolerability of antifungal drug even with the lower dose of therapeutic ingredient when applied topically.^149–151^ For improved topical drug delivery, NEs are suitable substitutes for lipidic vesicular carriers, including liposomes, noisomes, phytosomes, *etc.*

NEs, as transungual delivery carriers, have come into view for the onychomycosis treatment, providing beneficial therapy without any side effects. A nanoemulgel can be formed by converting NEs into a gel. This provides added stability and prolongs the drug's contact time with the affected area. By incorporating penetration enhancers, such as thioglycolic acid, the permeation of the drug through the nail plate can be further enhanced. Mahtab and co-workers further developed 55 NEs for the delivery of ketoconazole and the optimized emulsion as nanoemulgel for the transungual delivery of the drug—the TEM images of NEs revealed the spherical shape of globules. The drug release from NEs (NE3), nanoemulgel (NEG1), and drug suspension was evaluated in phosphate buffer media using a dialysis membrane, showing a significant difference in drug release. The permeation was performed utilizing the membrane of the goat hoof. The NEG1 showed maximum permeation compared to NE3 and drug suspension. Comparative antifungal activity was performed on *C. albicans* and *T. rubrum*. After 48 hours of study, the ZOI was found to be approximately similar for both fungal strains in the case of NEG1, which is comparatively higher than the ZOI observed by applying drug suspension.^152^ A study reported by Fatima and coworkers elaborates on the development of NEs of amorolfine, and then nanoemulgel was prepared for optimized formulation utilizing thioglycolic acid as a permeation enhancer. The films developed (Fig. 4-V) for drug-free dual-component loaded nail lacquer contain HPC (5% w/v), Eudragit 100 RS (5% w/v), and ethanol : water (9 : 1) ml. The drug release from NEs-based nail liquor was observed in PBS and showed better sustained release than in the marketed formulation; the release kinetic study revealed formulations that followed the Higuchi model. The transungual permeation was evaluated on the human nails; the nanoemulgel showed improved permeation than the marketed formulation.^145^

#### 7.2.3 Solid lipid nanoparticles (SLNs)

SLNs became a viable alternative delivery vehicle because of their many advantages over liposomes, including integrating hydrophilic and lipophilic drugs, low skin irritation, improved stability for up to three years, controlled release, and lower costs.^153–156^ When they dry, SLNs form a film on the skin surface or nail, preventing water loss and enhancing delivery.^157^ This occlusion effect is not specific to SLNs but may be more pronounced because of their nanoscale size. The large surface area of SLNs allows for more contact between the drug and the SC and may also lead to prolonged retention of the drug in the skin layers, resulting in sustained release.^158^ NLCs have been developed due to some drawbacks of SLNs, like aggregation, relatively low drug loading, and storage stability. Tiwari and coworkers reported the development of a terbinafine hydrochloride gel formulation based on SLNs. SLNs were incorporated in chitosan gel and evaluated for antifungal activity and drug release. Terbinafine hydrochloride release from SLNs and their gel formulation confirmed sustained release. The SLN-based formulation enhanced antifungal efficacy against *C. albicans* compared to the marketed formulation.^159^ Abobakr *et al.* developed and characterized SLNs for the onychomycosis treatment where terbinafine hydrochloride was used as an antifungal agent. The SLN formulation was comparatively evaluated with the marketed cream Lamifen^®^. The *in vitro* antifungal activity of the SLN formulation showed a more significant zone of inhibition and significantly higher drug uptake than that of the marketed formulation. N_2_ showed 1.78 times while N8 showed 1.67 times ZOI, depicting significantly higher antifungal activity compared to marketed Lamifen cream. The SLN formulation also improved nail hydration, a critical requirement in onychomycosis treatment.^160^

#### 7.2.4 Nanostructured lipid carriers

**Fig. 5:**
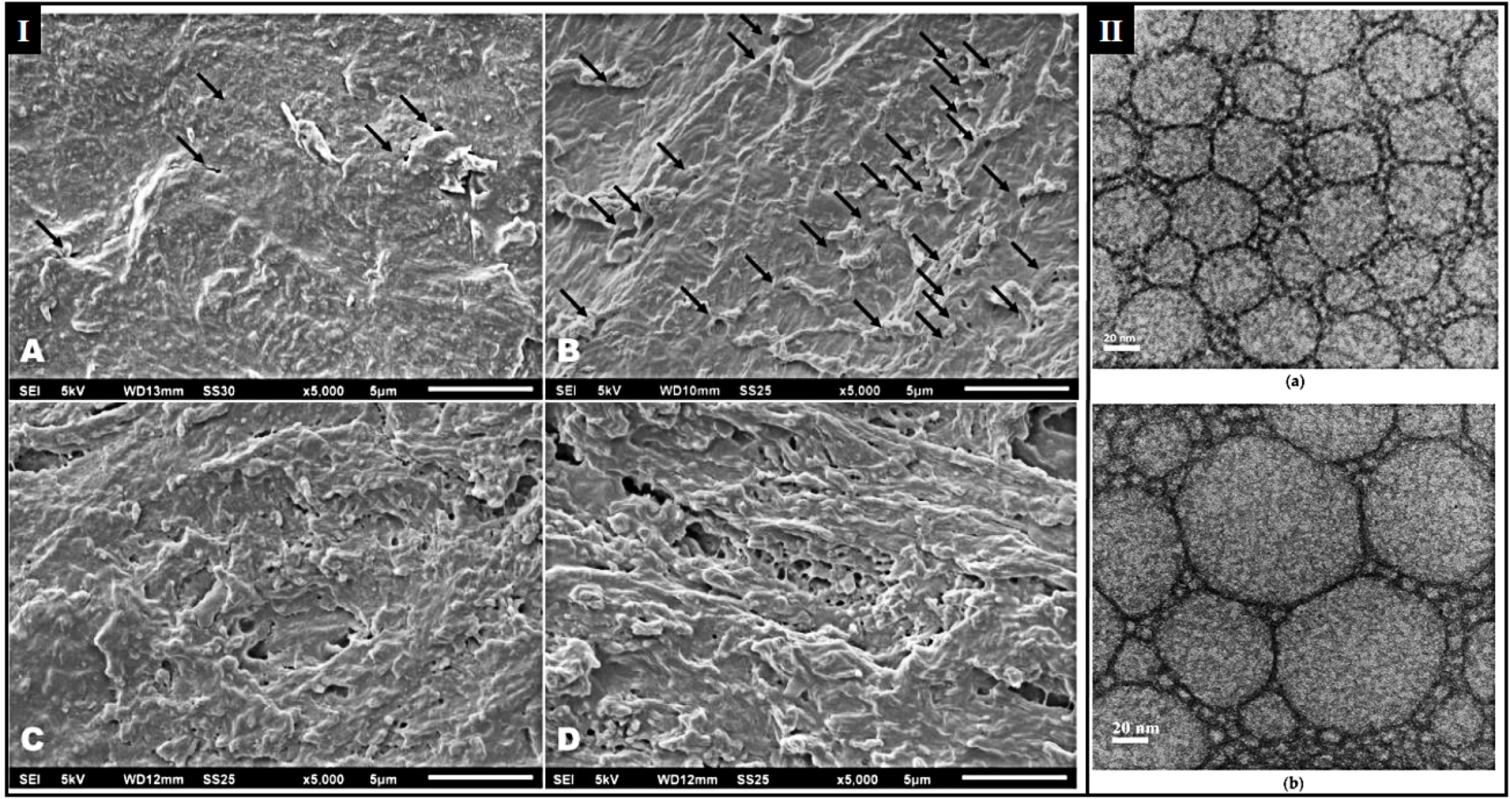
(I) SEM of surface of porcine hoof – (A) untreated; (B) water treated (C) with VOR-NLC treated; (D) VOR-NLC-Ur treated (this figure has been reproduced from ref. Rocha *et al.*^167^ with permission from Elsevier, copyright 2017); (II) TEM the NLC formulations (a) F 30 and (b) F 85 (the figure adapted from Pereira *et al.*^168^ under CC BY license).

NLCs are second-generation lipid nanocarrier systems derived from o/w nanoemulsions. The NLCs comprise a blend of solid and liquid lipids that are biodegradable and compatible with each other and the drug.^161^ Using liquid lipids helps reduce the melting point of solid lipids and avoids the recrystallization of solid lipids. The liquid-to-solid lipid ratio can vary from 30 : 70 to 0.1 : 99.9. The NLCs have comparatively high drug loading and payload of lipophilic and hydrophilic drugs and prevent drug leakage from the NLCs matrix. The NLCs are stabilized in aqueous dispersion using an optimized surfactant concentration with or without co-surfactant.^162,163^ NLCs are categorized into three categories. NLCs are classified into three types based on their structure and composition. Type I, or imperfect crystal NLCs, are formed by mixing solid and liquid lipids, resulting in an imperfect lattice that allows higher drug loading by avoiding a highly ordered matrix. Type II, known as amorphous or structureless NLCs, are composed of medium-chain triglycerides with solid lipids, creating an amorphous core where drugs remain embedded, thus preventing crystallization and improving drug stability. Type III NLCs, or multiple types, are formulated by mixing solid lipids with oils, forming nano-compartments within the matrix, and utilizing the w/o/w emulsion concept to enhance drug loading capacity and stability.^164–166^ The investigation undertaken by Rocha *et al.* reported the development of NLCs utilizing urea as a permeation enhancer for delivering VOR. The *in vitro* permeation was tested on porcine hooves. The structural changes observed are represented in Fig. 5-I. Hooves without treatment show a smooth surface (Fig. 5-IA), whereas hydration (Fig. 5-IB) increased keratin expansion, leading to irregular surfaces and pores due to the weakening of van der Waals forces, hydrogen bonds, and ionic interactions. All these processes are further disrupted with VOR-NLC and VOR-NLC-Ur in Fig. 5-IC and ID, further increasing the roughness and porosity due to the destabilization of ionic interactions by cetylpyridinium chloride in keratin.^167^

Remarkably, successive extractions revealed a considerable increase in the concentration of VOR retained in deeper regions when VOR-NLC or VOR-NLC-Ur was used, suggesting the potential of NLCs for treating onychomycosis.^167^ Research conducted by Pereira *et al.*, the authors reported optimizing and developing Ucuùba fat-based NLCs for delivering ketoconazole. The drug encapsulation was observed above 90%. Two optimized formulations with particle sizes 30 nm and 85 nm were preferred and characterized up to 30 days of storage at room temperature; the TEM photographs are depicted in Fig. 5-II, showing the spherical shape. Formulations remained stable over time despite initial drug loss during pre-emulsification and homogenization. The drug release of free drug solution and F 30 and F 85 were evaluated up to 24 h. NLC confirmed the controlled release of ketoconazole, with F 30 and F 80 releasing 81.3% and 45.0% of the drug after 24 hours, respectively. The lipid phase composition influenced particle size, PDI, drug entrapment, and drug release rate. The variation in the release was due to the variation in the composition of both formulations.^168^

#### 7.2.5 Liposomes

Liposomes, as vesicular vehicles composed of phospholipids, play a considerable role in enhancing the drug(s) pervasion through the upper layer of the stratum corneum. Despite their efficacy in topical drug delivery, liposomes' limited ability to penetrate the skin's deeper layers constrains their use as transdermal delivery systems.^169,170^ Specifically, nanosized liposomes are employed as topical drug delivery systems, where their efficacy relies on several factors, including size, lipid and cholesterol content, ingredient percentage, lamellarity, and surface charge. Such characteristics allow liposomes to tailor their functions in topical drug delivery. Liposomes in the topical drug delivery system are attributed to their amphiphilic nature, differentiating them as excellent permeation enhancers for topical administration compared to conventional formulations.^124,171,172^ Due to their physicochemical properties and safety profiles, liposomes have been widely used to treat microbial infections. Liposomes comprise one or more phospholipid layers that encapsulate the aqueous phase. They can be surface engineered using polymer, targeting ligands, *etc.*, to make them suitable for desired antifungal drug delivery applications. Liposomes can treat infections related to dermatophytes, including *T. rubrum* and *C. albicans*, and provide numerous benefits over conventional treatment by overcoming the emergence of resistant microorganisms and concerns associated with the drug's efficacy. They can be surface engineered using polymer, targeting ligands, *etc.*, to make them suitable for desired antifungal drug delivery applications. Liposomes can treat infections related to dermatophytes, including *T. rubrum* and *C. albicans*, and provide numerous benefits over conventional treatment by overcoming the emergence of resistant microorganisms and concerns associated with the drug's efficacy. Treatments for nail disorders may become more effective when liposomes, which are lipophilic in nature, utilize the lipophilic pathways in the nail plate.^173^ Several antifungal therapeutic agents have previously been delivered using liposomes, including itraconazole, amphotericin, ketoconazole, *etc.* The empirical work conducted by Tuncay and colleagues documented their findings on the ability of liposome-loaded Eudragit films (LI-E) and pullulan films (LI-P) formulations to deliver terbinafine hydrochloride. It was characterized by antifungal activity in onychomycosis. Liposome film (LI-P), a thinner film formulation, showed comparatively high and faster drug release than other formulations. *Ex vivo* nail permeations were evaluated using the TOWL study. The TOWL of the nail was found to be increased due to swelling in each case. The lowest value was noted because the LI-P formulation had less impact on the nail. The day after the experiment, the nails dried, and the TOWL values returned to baseline. Nail permeation revealed drug accumulation in the nail, and after ten days, the drug was not found in the receptor compartment, confirming the potential of formulations for the first time; an *in vivo* antifungal experiment was executed to study the therapeutic efficacy of these preparations against *T. mentagrophytes* infected nails. After 5 weeks of infection, the fungal hyphae and spores were visible within the plate. After 6 weeks of treatment, all formulations showed considerable therapeutic effects; rarely, hyphae and spores were seen after treatment. Residual hyphae persisted with ethosome and ethosome chitosan gel formulations, but lesser spores persisted in liposome and liposome poloxamer formulations. The best result came with the formulation (LI-P), in which neither hyphae nor spores were detected.^174^ Shah and collaborator prepared liposomal formulation of nail lacquer of terbinafine HCl by optimizing them with the response surface method using BBD. Transungual permeability flux across human nail plate was notably higher in the case of novel liposomal formulation than nail lacquer comprising permeation enhancer. At the same time, similar results were observed in the case of cattle hoof, improving therapeutic efficiency. The higher permeation rate of the liposomal formulation was attributed to small vesicle size, hydration forces, and osmotic gradient. Antifungal activity confirmed that the formulation's efficacy was comparable to that of the pure drug solution.^175^ A comprehensive study by Tuncay, 2018 reported their observations on transungual drug delivery using liposomes and ethosomes to evaluate their permeation capabilities. The particle size of liposomes was found to be relatively higher than that of the ethosomes. The *ex vivo* permeation studies performed on human cadaver nails revealed the potential of ethosomes, which showed comparatively more permeation through nails than liposomes. The permeation results confirmed the potential of both formulations to enhance the drug permeation.^173^ Chouhan *et al.* (2021) developed nail liquor loaded with liposomes to deliver antifungal luliconazole. The liposomal suspension (LF) was prepared using a modified film hydration technique. The liposomal suspension showed maximum drug release compared to nail liquor. The antifungal activity against *C. albicans* showed a maximum of 47 mm ZOI compared to plain nail liquor of luliconazole, which showed 36 mm of ZOI.^176^

#### 7.2.6 Invasomes

**Fig. 6:**
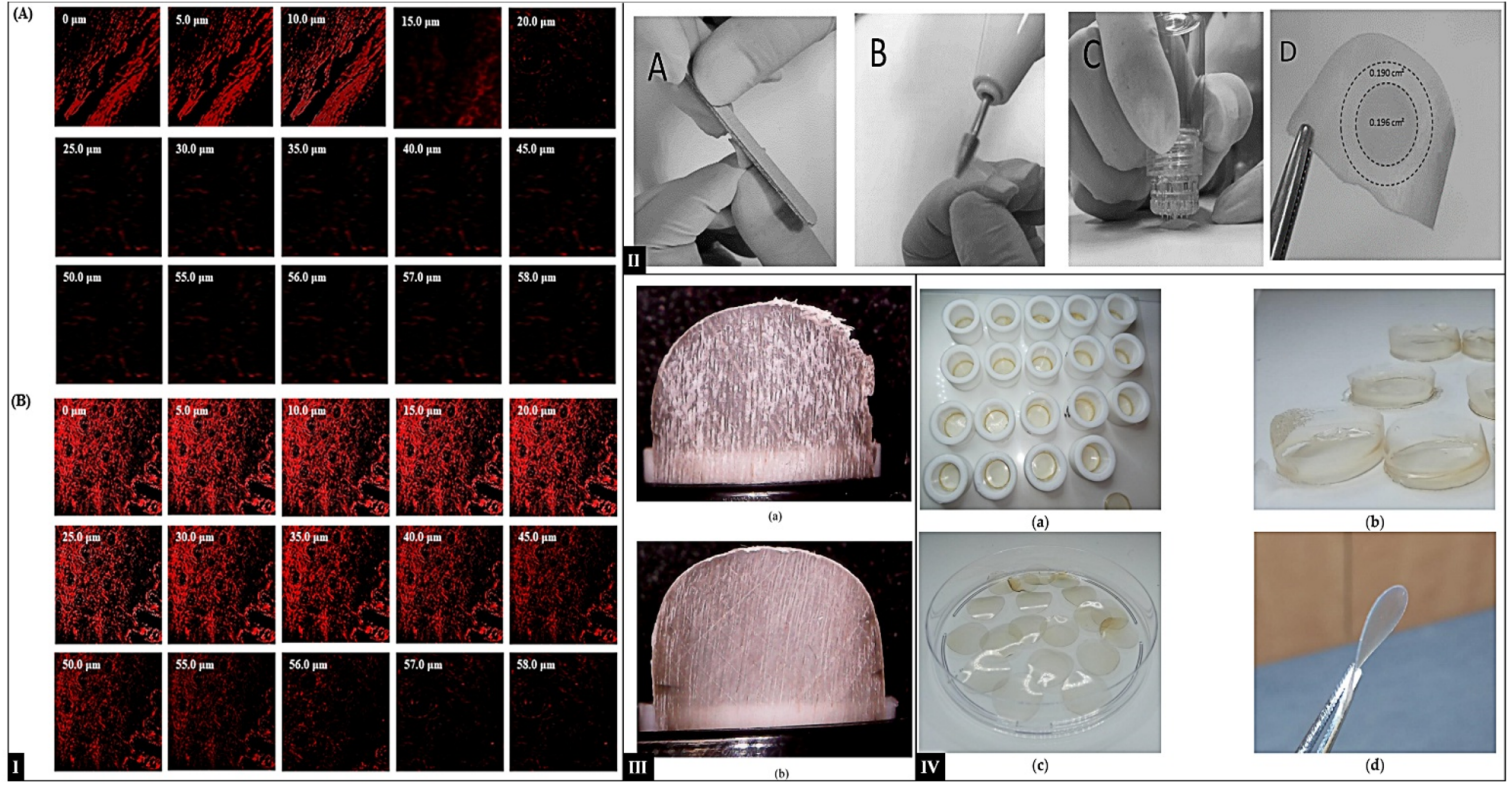
(I) CLSM images of goat hooves (A) TBF suspension treated and (B) TBF-INopt treated; (the figure adapted from Gupta *et al.*^183^ under CC BY license); (II-A) manually sanding of nails; (II-B) microporation by electric filing; (II-C) poration using Hydra needle; (II-D) after IVPT nail sections to quantify CPO in the diffusional area and the outer ring (the figure adapted from Kishishita *et al.*^184^ under CC BY license); (III) comparison of membrane formed: (A) flaky membrane; (B) smooth membrane (this figure has been reproduced from ref. Bonetti *et al.*^185^ with permission from Elsevier, copyright 2021); (IV) keratin films: (a) intermediate soft KF; (b) KF-40 before punching; (c) KF-40 after punching; and (d) KF-110 after curing (the figure adapted from Valkov *et al.*^186^ under CC BY license).

Invasomes represent a class of modified liposome carriers intended to improve drug penetration significantly. Liposomes have a rigid membrane, consist mainly of cholesterol and phospholipids, and have limited penetration into the skin. Structurally, invasomes consist of a bilayer membrane composed of soy phosphatidylcholine, lysophosphatidylcholine, terpenes, and ethanol.^177–179^ Each component specifically optimizes delivery efficiency: phosphatidylcholine acts as the primary bilayer-forming lipid, while lysophosphatidylcholine confers the membrane flexibility critical for adaptation to the skin microenvironment. Ethanol and terpenes act as powerful permeation enhancers, increasing the fluidity and elasticity of the bilayer, parameters directly linked to improved intradermal penetration capacity. Ethanol further contributes to the physicochemical stability of invasomes by inducing a strongly negative surface charge, effectively reducing vesicle aggregation *via* electrostatic repulsion.^180,181^ This stabilization minimizes vesicle fusion during storage, maintaining vesicle integrity and optimizing shelf-life. The unique compositional framework of invasomes enables efficient and sustained drug release and improves transdermal bioavailability, underscoring their utility in therapeutic interventions requiring enhanced ungual and dermal absorption.^181^ Hoda and collaborators developed valencene-modified invasomes to potentiate the transungual delivery of itraconazole (ITZ), with optimization conducted *via* the CCD approach. The ITZ-loaded invasome gel demonstrated exceptional homogeneity, with a viscosity of 7.33 ± 0.67 Pa s, and drug EE of 94.13 ± 1.13%. Spreadability and extrudability measurements yielded values of 7.85 ± 0.24 g cm s^−1^ and 162 ± 2.74 g, respectively, indicative of the gel's optimal rheological and application properties. Permeation studies using goat hoof membranes exhibited a cumulative permeation of 71.11 ± 3.65%, signifying superior penetration efficiency of the ITZ-loaded invasome gel across keratinized substrates. *In vitro* antifungal activity assays against *T. rubrum* revealed a significantly larger zone of inhibition for the invasome formulation (21.42 mm) compared to a commercial itraconazole gel (10.64 mm), underscoring its potent fungicidal activity. The enhanced efficacy is likely attributed to the invasome's ability to facilitate ITZ vesicular diffusion through fungal cell walls, consequently inhibiting ergosterol biosynthesis more effectively.^182^ To explore the potential of invasomes in transungual delivery, Gupta and colleagues developed linalool-containing invasomes for terbinafine (TBF-IN) delivery and optimized the formulation using the BBD approach. The optimized invasome formulation (TBF-INopt) showed a spherical sealed vesicle and EE of 74.23%. CLSM showed that TBF-IN penetrated the keratin layer of goat hooves to a depth of 56 μm, significantly more profound than the control TBF solution, which only reached 15 μm. Furthermore, the TBF-INopt formulation showed higher fluorescence intensity than the TBF suspension, indicating a more uniform distribution of TBF in deeper hoof layers, confirming improved drug permeation (Fig. 6-I). The TBF-INopt gel demonstrated significantly increased antifungal activity compared to the marketed terbinafine gel, as evidenced by a greater ZOI against both *T. rubrum* and *C. albicans*. In particular, the TBF-INopt gel demonstrated a 1.6-fold larger zone of inhibition against *T. rubrum* than the marketed gel. Against *C. albicans*, the TBF-INopt gel demonstrated a 2.3-fold increase in the zone of inhibition compared to the marketed gel. The control vehicle showed no inhibitory effect against any of the microorganisms. This suggests that the TBF-INopt gel formulation provides significantly superior antifungal efficacy.^183^

#### 7.2.7 Transferosome

Transferosomes are deformable and stress-responsive lipid-based vesicles comprising phospholipids and edge activators. Edge activator is a membrane softening agent that helps to enable the ultra-deformable attributes and increases the lipid bilayer flexibility and permeability of the transferosome.^187,188^ Such properties allow transferosomes to dehydrate and deform to permeate even across the tiny pores and then recover their shape through the rehydration process.^189^ Driven by the transcutaneous water gradient, transferosomes can penetrate the permeability barrier of skin and nails.^190^ A study reported the potential of nanovesicles for *trans*-ungual drug delivery. The ethanol injection approach was used in the development of the formulation, optimized using response surface methodology, and evaluated for *ex vivo* nail permeation study. The permeation and retainment were assessed on a cadaver nail plate for 03 days, and the nanovesicle permeation was higher than the marketed formulation.^191^ Another study reported the liquid spray-based transfer formulation for terbinafine (TDT 067) delivery. The formulations were evaluated for their antifungal efficacy against 25 strains. The MI_50_ value of TDT 067 was 60-fold lower than terbinafine spray and eight-fold lower than free terbinafine solution. *In vivo* study of the morphological effects of TDT 067 in patients with onychomycosis, assessed through subungual samples following 28 days of topical administration. TEM analysis demonstrated significant morphological alterations, including the formation of large lipid droplets within fungal cells as early as 7 days, supporting *in vitro* observations. TEM revealed that TDT 067 induced rapid disruption of intracellular structures, with detectable changes evident within 5 minutes of exposure. Moreover, the investigation showed that terbinafine concentrations in nail debris were markedly lower post-treatment than those retained with oral terbinafine, indicating reduced binding affinity within the nail matrix. The findings emphasize TDT 067 capacity to effectively penetrate nail tissue and exert rapid antifungal activity, which may account for the elevated mycological cure rates observed in Phase-II open-label efficacy trials.^190^

#### 7.2.8 Niosomes

Niosomes are a multifaceted vesicular vehicle composed of cholesterol and nonionic surfactants. Structurally, these are bilayered carriers wherein the hydrophilic head is exterior and interior-centric, whereas the hydrophobic tail resides between the hydrophilic heads. Therefore, the arrangements of components facilitate niosomes to deliver lipophilic drugs *via* lipid material and hydrophilic drugs *via* an aqueous core.^169^ Niosomes contribute several advantages, covering high drug loading ability, better stability, scalability, and lower formulation cost than liposomes.^192^ However, such properties depend on various conditions, including the formulation method, lipids concentration, and other ingredients used.^192,193^ Additionally, niosomes are osmotically active, non-toxic, non-immunogenic, biocompatible, and biodegradable. The functionality can be improved by overcoming biological complexity and therapeutic challenges associated with the niosomes. The niosomes can be formed multifunctionally by modification with targeting ligands, stimuli-responsive moiety, polyethylene glycol, *etc.* The multifunctional niosomes show improved therapeutic ability, which is attributed to the synergic benefits of linking several targeting mechanisms.^194^ A study reported the delivery of efinaconazole utilizing niosomes-based nail liquor. The developed niosomes were smaller in size, ranging between 95 nm to 135 nm. The niosomes-based liquor showed comparatively significantly more drug release than other formulations.^195^ Another study reported the antimycotic activity of itraconazole-loaded niosomes. The niosomes were tested for size, drug release, and antimycotic activity. The niosomes showed good permeation through the skin and were found to show comparatively improved antimycotic activity against *C. albicans* than that of the marketed formulation.^196^

## 8. Nail models for evaluating drug penetration

The permeability of drugs through the nail plate is assessed using various *in vitro*, and *ex vivo* models. To evaluate drug transport under controlled circumstances, *ex vivo* models use human or animal nails mounted in diffusion cells, whereas *in vitro* models usually use artificial membranes that mimic nail characteristics. Lipid-based nanocarriers, due to their ability to hydrate and disrupt intercellular lipid domains, facilitate deeper penetration, which is particularly useful in cadaver nail models where drug transport is inherently slow. These nanocarriers promote prolonged drug retention within the nail matrix, improving therapeutic outcomes in antifungal and antimicrobial treatments. Their adaptability to alternative models, such as bovine hoof membranes, compensates for differences in keratin density and disulfide bond content, ensuring comparable permeation kinetics. In human nail studies, inter-donor variability in thickness and composition can lead to inconsistent results, but lipid nanocarriers provide controlled and reproducible drug release, reducing variability. The slow drug diffusion across the nail necessitates highly sensitive analytical tools, and lipid nanocarriers improve drug solubilization, aiding in precise detection and quantification. Additionally, they enhance the bioavailability of poorly soluble drugs, ensuring higher therapeutic concentrations at the site of action. Their biocompatibility makes them ideal candidates for long-term drug delivery applications with minimal toxicity concerns. These advantages collectively improve the accuracy and reliability of drug permeation studies across different nail models, making lipid-based nanocarriers a valuable tool in transungual drug delivery research.

### 8.1 Human nail plate models

Acquiring human plates can be challenging, as nails are hardly available after nail loss or surgeries. If obtained, nail plates from different donors can vary in composition, thickness, and penetration kinetics, leading to less credible data. The tiny size and curved shape make it difficult to mount them on standard diffusion cells. Besides, drug transport through nails is typically slow and limited, necessitating compassionate, analytical tools to detect and quantify the minute amounts of drug permeating. While human nail plates offer valuable insight into transungual drug delivery, their limitations necessitate exploring alternative models and analytical techniques to enhance the accuracy and efficiency of drug permeation studies.

### 8.2 Cadaver nail

The cadaver nail provides the closest anatomical structure of a human nail and the actual dynamics of therapeutic agent permeation through the nail plate. The benefit of using this model is its ability to replicate the different nail conditions (like structural traits, thickness, and keratin content) of the patient with onychomycosis. They can be directly infected with fungi like *T. rubrum*, enabling research on antifungal agent efficacy within the actual disease context. However, cadaver nails necessitate ethical considerations, may require specific regulatory approvals, and can cause difficulties. Moreover, individual variations in donor health and age impact nail properties, leading to potential inconsistencies in data.^197^ Fingernails from corpses can be purchased from hospitals or tissue banks. They had to be stored at −80 °C.^198^ Tuncay (2018) developed liposomal and ethosomes formulations for transungual drug delivery. Human cadaver nails were used as a model in the *ex vivo* investigations to assess their penetration. The nail plates were allowed to acclimate to room temperature overnight. The nail plates were weighed, and their thickness was measured before the permeation experiments commenced.^173^ Kishishita and a coworker procured the human fingernail clippings with ethical approval, which were 8 mm long or more. The clippings were kept for further preparation for *in vitro* permeation IVPT and microbiological tests at −20 °C. Before performing the tests, two treatments were employed: non-prorated nails were soaked in water for 2 hours, and slight sanding was done, whereas prorated pores nails were also soaked in water, were sanded using an electric file, and microporated using a device with 0.60 mm titanium needles creating approximately 20 pores for testing (Fig. 6-IIA–C). In attempts to measure nail plate permeability, nails were swabbed with isopropyl alcohol. The central circular area of the diffusion region (Fig. 6-IID) was cut off, and the cut portions were used for the investigations. The cut samples were transferred into centrifuge tubes containing seventy percent ethanol and left in a shaking water bath for seven days. After that, the liquor was separated, and the samples were examined using the HPLC-UV method. To investigate the lateral transport of active substances, a ring of the surrounding nail cut off the diffusion region was also evaluated this area of diffusion.^184^

### 8.3 Bovine hoof membrane

Nails can be replaced by hooves in the studies that deal with permeation characteristics. Hooves, however, are, on average, lower than human nails in terms of periungual penetration enhancers. They are more permeable than human nails because of their relatively lower number of disulfide bonds.^135^ Bovine hoof membranes are models in permeability studies as these mimic natural nail conditions. While using Bovine membranes as a model, it is crucial to consider the differences between the bovine hoof membrane and the human nail plate.^110^ The primary source of hoof membranes is freshly slaughtered cattle. The hoof is typically soaked in water after the surrounding tissue has been removed, and membranes are then cut with a microtome to achieve the appropriate thickness. Since they are byproducts of slaughterhouses, bovine hooves are usually available and typically inexpensive or free, but human nails are difficult to source and costly. The potential of this model for applications other than permeability studies remains unexplored. The bovine hoof membranes have a less dense/porous keratin network, leading to less resistance to drug permeation. Incubation in water causes hoof membranes to swell up 36%, compared to 27% for human nail clippings.^185,199^ Furthermore, compared to human nails, hoof proteins probably contain fewer disulfide bonds and significantly fewer cysteine residues. Therefore, enhancers such as breaking keratin disulfide bonds on bovine hooves and nail plates can exhibit varied effects.^199^ Bonetti and their coworker worked on hoof membranes' production protocol and characterization. Hoof membranes from freshly slaughtered 3 years cattle were obtained, immersed in liquid nitrogen, and then coring with a 16 mm plug cutter, followed by slicing on a precision lathe, were cleaned with a 70% v/v ethanol solution and mix of benzalkonium chloride and isopropyl alcohol followed under controlled conditions for 3 days. The membranes (Fig. 6-III) were further classified as flaky, smooth, or borderline based on their morphological features, surface desquamation, and thickness.^185^ Temporiti and coworkers reported that membranes were acquired from freshly slaughtered cattle. The resulting hooves were soaked in liquid nitrogen to avoid deformation during core drilling and cutting. They were then cored with a plug cutter and cut into 800 μm long slices using a precision lathe. In their experiment, 28 membranes were chosen and sanitized using ethanol 70% v/v and a mixture of benzalkonium chloride, isopropyl alcohol, and water (0.4 g, 70, and 100 g distilled water, respectively). Membranes were kept at 25 °C and 40% RH. After that, the prepared membrane's mechanical characteristics and contact angle were assessed. Since the developed membrane proved successful, membranes with the appropriate mechanical properties, consistent morphology, and optimal thickness could be produced.^200^

### 8.4 Keratin biomembranes

These are artificial films produced from keratin, the critical constituent of human nails.^201^ They are usually prepared by extracting keratin from human hair and then reconstituting it under specific conditions. This results in membranes with structural and compositional similarities to natural nail plates.^202,203^ Compared to actual human nails, keratin bio-membranes are ethically obtained and available in consistent sizes, providing a controlled environment for assessing drug permeation. The composition and thickness of the keratin film can be adjusted to mimic different nail types and conditions, allowing researchers to conduct reproducible and reliable measurements and enhancing the model's accuracy.^204^ Valkov and colleagues reported a method for producing keratin films from human hair that can be used as a model for nail permeability study. The technique involves extracting keratin from human hair using a 1,4-dithiothreitol solution. The extracted keratin was filtered, dialyzed, and centrifuged to remove aggregates. The resulting solution was then dispensed into Teflon rings, and water was evaporated to form soft keratin films. The films were then punched and cured at 110 °C to form rigid keratin films Fig. 6-IV. The width of the films formed was approximately 67 μm. In FTIR investigations of human hair, nails, KF-110, and bovine hoof samples, characteristic absorption bands are amide A, I, II, and III. Samples of nails invariably have sensitive peaks of amide A centered at 3295–3390 cm^−1^ shifted due to hydrogen bonds with humidity in the samples. The films of human hair and KF-110 have identical spectra. Films of human hair and KF-110 miss one or two bands from nail-specific ones. Therefore, keratin films could be considered a good model for nails.^186^ While promising, keratin biomembranes still have limitations. They may not fully capture the complexity of the natural nail environment, and scaling up production for large-scale studies can be challenging.

## 9. Onychopharmacokinetics of antifungals

Onychopharmacokinetics focuses on understanding the dynamics of drug permeation through the nail and the factors influencing drug retention and efficacy in treating nail disorders (such as onychomycosis). Key factors influencing onychopharmacokinetics include the drug's molecular size, lipophilicity, formulation vehicle, and the condition of the nail itself, as any damage or alteration in the nail structure can affect drug penetration and distribution. Since the treatment efficacy is a function of the capability of topically applied antifungal agents to penetrate the strong keratin matrix of the nail and directly reach the infection site, different research studies have established a good correlation between the ability of the drug to permeate the nail and clinical efficacy in infections such as onychomycosis. PK/PD metrics include the *f*AUC/MIC ratio, *f*AUC for the unbound, free drug, and the efficacy coefficient proposed as a marker of clinical effectiveness during antifungal treatment. On this basis, through relationships established between PK/PD measures and clinical results, professionals and researchers can predict the success of therapy more accurately and improve dosing schedules with better patient outcomes in addressing fungal infections.^205,206^ Hui *et al.* examined the onychopharmacokinetics of the novel antifungal drug “ME1111” using a finite dose *in vitro* model. Compared to the MIC90 and MFC90 for major dermatophytes, ME1111 concentrations were higher in the deeper nail and cotton pad/nail bed. At 3 days after a single dose and 14 days of multiple doses of 10% ME1111, concentrations reached 253 and 7991 μg g^−1^ in nails, respectively, exceeding the values of 8% ciclopirox. ME1111 concentrations in the cotton pad increased linearly throughout the 336 hours experiment and were much higher than ciclopirox. With a flux rate of 50.9 μg per cm^3^ per day, ME1111 demonstrated superior penetration into the nail plate, significantly exceeding ciclopirox levels.^207^ Jarratt and coworkers examined the pharmacokinetics of a 10% efinaconazole solution and assessed potential DDI when applied topically to patients with onychomycosis and healthy volunteers. The mean *C*_max_ of efinaconazole was 0.54 ng ml^−1^ in healthy volunteers and 0.67 ng ml^−1^ in patients. Its metabolite H3 reached 1.63 ng ml^−1^ and 2.36 ng ml^−1^, respectively. Both accumulated after repeated dosing, reaching a steady state by day 14. Efinaconazole showed very low systemic exposure and was well tolerated without adverse events. Despite being a CYP inhibitor, the *C*_max_/*k*_*i*_ ratio of 0.007 suggests that efinaconazole 10% solution has a low potential for clinically significant DDIs.^208^ Finlay studied terbinafine permeation in 12 patients over 48 days with a 250 mg per day dose. Terbinafine levels of 0.25–0.55 ng mg^−1^ were quickly reached and remained stable, with similar concentrations in affected and unaffected nails. While these levels fall within the fungicidal range, the anatomy of infected nails may remain protected, leading to persistent or recurrent infections.^209^ In another study, Kubota-Ishida *et al.* studied the PK and PD of “ME1111”. In a 14 days *in vitro* model, ME1111 showed considerably higher concentrations in nails and cotton pads, unlike efinaconazole, ciclopirox, and amorolfine, similar to TVB. Deep nail layers and pads had free drug levels that were noticeably higher than *T. rubrum* MIC_90_. In contrast to other antifungals, ME1111 activity was not impacted by mild acidity (pH 5.0) or 5% human keratin. The antidermatophytic efficacy coefficient of ME1111 was substantially higher than that of other antifungals, both at pH 5.0 and pH 7.0.^210^ Krishna *et al.* explored the PK of POS in 146 adults with toenail onychomycosis. Participants received 100 mg, 200 mg, or 400 mg of POS once daily for 24 weeks or 400 mg daily for 12 weeks. POS concentrations in the toenails increased dose-dependently starting from week 2, with a toenail-to-plasma concentration ratio of roughly 3 : 1 by the end of the 24 weeks treatment at 400 mg. The study included all participants with baseline PK data and at least one posttreatment assessment. POS plasma concentrations increased with dose and remained steady once they reached a flat profile in the toenails. The drug was detectable from week 2 in the 200 mg and 400 mg groups, and concentrations continued to rise even after treatment ended, while plasma concentrations, as expected, declined after treatment cessation. The maximum toenail concentration (*T*_max_) was reached between 36 and 42 weeks.^211^

## 10. Clinical progress

Clinical trials for onychomycosis have improved knowledge of the safety and antifungal effectiveness of different formulations and have produced encouraging outcomes in patient populations specifically targeted, such as children and people with comorbid conditions. When treating fungal infections of the toenails, topical medications like efinaconazole and TVB have shown long-term effectiveness and excellent tolerability, meeting the need for safer substitutes for systemic treatments. Longer treatment times, frequently necessary for complete nail restoration, showed that such therapies could be optimized for extended use without posing serious safety risks and offered crucial information on recurrence rates.

Pannu *et al.* (2009) in their clinical trial demonstrated that NB-002, an oil-in-water nanoemulsion-based antifungal, exhibits broad-spectrum fungicidal activity against major dermatophytes (*T. rubrum*, *T. mentagrophytes*, *Epidermophyton floccosum*, and *Microsporum* spp.), several filamentous fungi, and both azole-susceptible and azole-resistant *C. albicans*. Unlike conventional antifungal drugs such as ciclopirox, terbinafine, and itraconazole, which were largely fungistatic, NB-002 displayed rapid fungicidal activity, effectively killing both mycelia and microconidia, including dormant fungal forms. The study also found low spontaneous resistance development, with minimal MIC variation in resistant mutants, suggesting a lower possibility of resistance emergence than conventional therapies. Electron microscopy revealed severe structural disruption in fungal cells exposed to NB-002, supporting its kill-on-contact mechanism. These findings underline NB-002 as a capable topical antifungal agent with superior efficacy and resistance-limiting potential.^212^ Firooz and colleagues (2023) conducted a pilot clinical study in 15 subjects (18 to 60 years of age) to assess the efficacy and safety of topical onychomycosis-resistant micrograms of amphotericin B 0.4 mg gel. Patients applied the gel twice daily for up to 36 weeks and were monitored at 12, 24, and 36 weeks. By week 24, 91.66% of patients had achieved complete and mycotic recovery, with only 8.33% not responding to treatment. The most common adverse reaction was temporary discoloration of the nails, while no systemic adverse reactions were reported. One patient had a nail fall in week 2, but was fully cured. These findings highlight that nanoliposomal amphotericin B is a highly effective and well tolerated alternative to systemic antifungal therapy and justify further extensive studies.^213^ In another trial, Moazeni *et al.* (2024) conducted a randomized clinical trial to evaluate the efficacy of a *Zataria multiflora* nanostructured lipid carrier (Zt-NLC) gel for Candida-associated onychomycosis. The study included 40 volunteers, aged 30 to 60 years, who were randomly assigned to receive either Zt-NLC gel or placebo for four weeks. The results showed a 97.5% mycological cure rate in the Zt-NLC group compared with 75% in the placebo group. Clinical assessments indicated a cure rate of 87.5%, which was significantly higher than that of placebo (67.5%). Patients treated with Zt-NLC gel experienced faster symptom relief, reduced pain, and higher satisfaction (75%). Additionally, the formulation exhibited enhanced drug permeation, sustained antifungal activity, and no cytotoxic effects, thereby confirming its safety and tolerability. These findings indicate that the Zt-NLC gel is a promising and patient-friendly alternative for the treatment of onychomycosis.^214^

Table 3 summarizes different clinical trial studies of the formulations for onychomycosis.

**Table 3:** Summary of phase 3 and 4 clinical trials for onychomycosis treatments

NCT number	Formulation type	Interventions	Brief summary	Enrolment	Sponsor
**Drug(s) in clinical trial for onychomycosis completed phase 4**					
NCT03110029	Solution	Efinaconazole 10% topical application solution, application of nail polish	This trial was evaluated using efinaconazole solution (Jublia) to treat toenail fungal infections in patients with and without nail polish	13	University of Alabama at Birmingham
NCT03280927	Solution	Jublia®	Jublia® topical solution was tested for 48 weeks on patients with mild or moderate dermatophyte-induced onychomycosis to assess its antifungal effectiveness and safety	97	Dong-A ST Co., Ltd
NCT02464826	Solution	Nailprotex	The study was designed to demonstrate this product's efficacy in treating non-dermatophyte onychomycosis and chronic paronychia in Thai patients	19	Mahidol University
NCT03405818	Solution	TVB 5% topical solution	An open-label study tested TVB 5% topical solution on children and adolescents (ages 6 to 16) with toenail fungal infections. The solution was applied daily for 48 weeks after confirming eligibility. Safety and infection extent were monitored throughout, with additional assessments at 52 weeks	55	Pfizer
NCT03814343	Microemulsion	Amphotericin B in 30% DMSO, 30% DMSO	The purpose was to compare the safety and efficacy of amphotericin B in a 30% DMSO solution with that of a 30% DMSO solution alone in treating onychomycosis triggered by NDMs	19	Mahidol University
NCT03098615	Solution	Jublia (efinaconazole 10% topical solution)	The study examined the effects of Jublia on dermatophytomas, which were challenging to treat with other options	19	University of Alabama at Birmingham
NCT02812771	Solution	Efinaconazole	The study assessed the safety and PK of daily topical efinaconazole for treating pediatric toenail onychomycosis	62	Bausch Health Americas, Inc.
NCT01419847	Nail lacquer	Ciclopirox, placebo	A five-month course of therapy effectively addressed onychomycosis in children. Penlac nail lacquer's topical treatment proved as effective as systemic therapy, offering superior cost-effectiveness and safety	40	Rady Children's Hospital, San Diego

**Drug(s) in clinical trial for onychomycosis completed phase 3**					
NCT02547701	Solution	P-3058	Pediatric patients with mild-to-moderate DSO or WSO caused by dermatophytes were treated topically with P-3058 nail solution according to the treatment schedule	20	Polichem S.A.
NCT02549001	Solution	P-3058 10%, vehicle of P-3058 10%, amorolfine 5%	The study tested whether the P-3058 nail solution safely and effectively treated onychomycosis	953	Polichem S.A.
NCT00781820	Cream	Bifonazole cream 1%, placebo cream	The study focused on demonstrating the superiority of 1% bifonazole cream over a placebo cream following non-surgical nail ablation using a 40% urea paste	693	Bayer
NCT00443820	Solution	Terbinafine, placebo, terbinafine, placebo	This study tested a daily topical terbinafine solution for toenail fungus, comparing 24 and 48 weeks treatments for mild to moderate cases on the big toe	526	Novartis Pharmaceuticals
NCT02859519	Solution	MOB015B, MOB015B vehicle	The study aimed to assess topical MOB015B's effectiveness and safety in patients with mild to moderate DSO	365	Moberg Pharma AB
NCT01007708	Solution	IDP-108, vehicle	The study aimed to compare IDP-108 and a vehicle in treating onychomycosis, evaluating both safety and efficacy	780	Dow Pharmaceutical Sciences
NCT03168841	Solution	Efinaconazole	Topical efinaconazole (10%) was tested for treating toenail onychomycosis in diabetic patients, and its efficacy and safety were assessed	40	Western University of Health Sciences
NCT01302119	Solution	AN2690 topical solution, 5%, solution vehicle	The study evaluated the safety and efficacy of AN2690 topical solution for treating toenail onychomycosis	604	Pfizer
NCT00459537	Nail lacquer	Terbinafine hydrogen chloride, amorolfine nail lacquer	The study compared 10% terbinafine applied daily to 5% amorolfine applied twice weekly for 48 weeks in patients with mild toenail onychomycosis	1029	Novartis
NCT02866032	Solution	MOB015B, Ciclopirox 80 mg g^−1^	The study aimed to assess topical MOB015B's effectiveness and safety in mild to moderate DSO patients	452	Moberg Pharma AB
NCT00443898	Solution	Terbinafine, placebo, terbinafine, placebo	The study evaluated a daily topical terbinafine solution with mild to moderate toenail fungus, comparing two treatment durations: 24 or 48 weeks	518	Novartis Pharmaceuticals
NCT01008033	Solution	IDP-108, vehicle	The trial aimed to assess the efficacy of IDP-108, which was compared to a vehicle when applied topically to treat patients with onychomycosis	870	Dow Pharmaceutical Sciences
NCT01270971	Solution	AN2690 topical solution, 5%, solution vehicle	The trial aimed to assess if the AN2690 topical solution safely and effectively treats toenail onychomycosis	594	Pfizer

## 11. Regulatory considerations

Nanomaterials are controlled similarly to any other regulated medicinal product, which implies that similar standards and regulations are followed to ensure their quality, safety, and efficacy. Nanomaterials play various roles in drug formulations, such as active ingredients, excipients, drug carriers, or complexes. Liposomes are the most common nanomaterial used, followed by nanocrystals, emulsions, iron–polymer complexes, micelles, and other materials like drug–protein and polymeric nanoparticles.^215^ nanoparticles may serve in roles such as drug carriers or combination products that integrate both drug and device and/or theranostic functionalities. The FDA has outlined specific guidelines to accurately classify whether a product falls under a drug or a device category. Section 201(g) of the FD&C Act (21 USC 321(g)) describes “drug” and section 201(h) of the FD&C Act (21 USC 321(h)) describes “device”. These guidelines are based on evaluating both the inherent properties of the product and its intended application.^216^ Despite their distinct nanoscale characteristics, which may result in unique biological interactions, regulatory authorities often adopt existing frameworks used for conventional drug products. This approach involves rigorous preclinical and clinical evaluation, toxicity testing, manufacturing controls, and post-market surveillance to monitor potential risks. Special consideration is given to the nanoscale size and surface properties, as these can influence absorption, distribution, metabolism, excretion, and potential environmental impacts. GMP regulations present significant concerns regarding developing and producing nano drug delivery systems to ensure quality, safety, and efficacy due to the unique nature of nanoparticles. Important concerns are maintaining sterility, which is difficult with such tiny particles, and accurately characterizing the size, shape, and drug encapsulation. Strict control over raw materials and process validation is needed to ensure consistency.^217,218^ Technology transfer presents notable challenges, mainly when the manufacturers are not the original developers of the process. In such cases, there can be a frequent lack of understanding regarding process performance, highlighting gaps that can emerge during the transfer process.^219^ To this end, the FDA guidance on drug products containing nanomaterials suggests the basis for assessing the safety, efficacy, and quality of such drug and biological products in which a nanomaterial plays an active role as an ingredient or carrier. The FDA further suggests monitoring nanomaterials' interaction with the biological system, particularly pharmacokinetics, biodistribution, and toxicity. Control over the manufacturing process will be essential to ensuring a quality product, whereas nonclinical and clinical studies should demonstrate how nanomaterials affect ADME. The FDA encourages a total case-by-case assessment to mitigate risks and ensure the safety and efficacy of nanomaterial-containing drug products.^220–222^

## 12. Conclusion and foresightful opportunities

Lipid-based drug delivery systems signify a growing approach to the treatment of onychomycosis, a disease that has proven to be persistent and difficult to treat effectively. Advanced lipid-based formulations have shown considerable potential to tackle significant treatment barriers, including MEs, NEs, SLNs, NLCs, liposomes, transfersomes, and niosomes. These enhance drug penetration, prolong the drug retention in the infected nail bed, and enable sustained delivery of antifungal agents directly to the site of infection. Clinical data shows that they can increase both nail permeation and therapeutic efficiency compared to conventional approaches, representing a promising option for treating this disease. Despite these advances, numerous challenges must be resolved to maximize the therapeutic potential of lipid-based systems. Optimization of formulation components, including the selection of lipids, co-solvents, and surfactants, remains critical to attaining desired physicochemical properties and compatibility with specific drugs. Scalability of manufacturing processes is also essential to confirm reproducibility and maintain product quality for large-scale production. Regulatory requirements for complex formulations add further complications and require robust safety, efficacy, and quality data to support clinical use. While the high penetration ability of these nanocarriers is beneficial for targeted drug delivery, it also poses the risk of systemic exposure if not adequately controlled. This concern is particularly relevant for antifungal therapies, where inadvertent systemic exposure could lead to side effects or drug resistance. Preclinical and clinical evaluations are essential to address these safety issues and ensure that formulations are practical and patient-friendly. In addition, surface modifications can be explored to increase stability, regulate drug release and optimize permeation rates, thus enabling a controlled and targeted therapeutic approach. By eliminating the limitations of current treatments, lipid-based drug delivery systems offer several advantages, including shorter treatment durations, improved patient adherence, and lower recurrence rates. Their nanoscale size allows them to penetrate nails and surrounding skin *via* mechanisms such as intercellular pathways, hydration-induced pathways and fusion with lipid bilayers, ensuring precise drug delivery to infected sites while minimizing systemic side effects. These innovative systems improve patient outcomes and reduce the overall burden on healthcare resources. Manufacturing and cost remain critical factors in the widespread adoption of these advanced systems. While their initial development costs may be higher, recent cost-effectiveness analyzes suggest that lipid-based therapies may reduce long-term costs.

## Abbreviation

BBD: Box–Behnken design
bioAgNPs: Biogenic silver nanoparticles
*C. albicans*
: *Candida albicans*
CLSM: Confocal laser scanning microscopy
DDI: Drug–drug interactions
EE: Encapsulation efficiency
*f*AUC: Area under the concentration–time curve
FD&C: Food drug and cosmetics
FDA: Food drug and administration
GMP: Good manufacturing practices
ICH: International Council for Harmonisation of Technical Requirements for Pharmaceuticals for Human Use
IVPT: *In vitro* permeation test
KF: Keratin films
LLLT: Low-level laser therapy
MEs: Microemulsion
MIC: Minimum inhibitory concentration
NDMs: Nondermatophyte moulds
NEs: Nanoemulsion
NLCs: Solid lipid nanocarriers
OEO: Oregano essential oil
OTC: Over-the-counter
PD: Pharmacodynamics
PDI: Polydispersity Index
PDT: Photodynamic therapy
PK: Pharmacokinetics
POS: Posaconazole
REO: Rosemary essential oil
RH: Relative humidity
SCIO: Scoring clinical index for onychomycosis
SEM: Scanning electron microscopy
SLNs: Nanostructured lipid carriers
*T. mentagrophytes*
: *Trichophyton mentagrophytes*
*T. rubrum*
: *Trichophyton rubrum*
TEM: Transmission electron microscopy
TOWL: Transbronchial water loss
TVB: Tavaborole
VOR: Voriconazole
ZOI: Zone of inhibition

## Data availability

No primary research results, no new data were generated or analysed as part of this review.

## Author contribution

Shiv Kumar Prajapati: conceptualization, data curation, methodology, investigation, formal analysis, writing – original draft. Meenakshi Bajpai: supervision, writing – original draft, writing – review and editing. Ankit Jain: data curation, writing – original draft, writing – review and editing, supervision.

## Conflicts of interest

The authors disclose no financial or non-financial interests that may affect the publication process for this manuscript.
